# A Pyroptosis-Related Gene Signature Predicts Prognosis and Immune Microenvironment for Breast Cancer Based on Computational Biology Techniques

**DOI:** 10.3389/fgene.2022.801056

**Published:** 2022-04-07

**Authors:** Zitao Wang, Anyu Bao, Shiyi Liu, Fangfang Dai, Yiping Gong, Yanxiang Cheng

**Affiliations:** ^1^ Department of Obstetrics and Gynecology, Renmin Hospital of Wuhan University, Wuhan, China; ^2^ Clinical Laboratory, Renmin Hospital of Wuhan University, Wuhan, China; ^3^ Department of Breast Surgery, Renmin Hospital of Wuhan University, Wuhan, China

**Keywords:** pyroptosis, gene signature, breast cancer, survival, tumor immune microenvironment

## Abstract

Breast cancer (BC) is a malignant tumor with high morbidity and mortality, which seriously threatens women’s health worldwide. Pyroptosis is closely correlated with immune landscape and the tumorigenesis and development of various cancers. However, studies about pyroptosis and immune microenvironment in BC are limited. Therefore, our study aimed to investigate the potential prognostic value of pyroptosis-related genes (PRGs) and their relationship to immune microenvironment in BC. First, we identified 38 differentially expressed PRGs between BC and normal tissues. Further on, the least absolute shrinkage and selection operator (LASSO) Cox regression and computational biology techniques were applied to construct a four-gene signature based on PRGs and patients in The Cancer Genome Atlas (TCGA) cohort were classified into high- and low-risk groups. Patients in the high-risk group showed significantly lower survival possibilities compared with the low-risk group, which was also verified in an external cohort. Furthermore, the risk model was characterized as an independent factor for predicting the overall survival (OS) of BC patients. What is more important, functional enrichment analyses demonstrated the robust correlation between risk score and immune infiltration, thereby we summarized genetic mutation variation of PRGs, evaluated the relationship between PRGs, different risk group and immune infiltration, tumor mutation burden (TMB), microsatellite instability (MSI), and immune checkpoint blockers (ICB), which indicated that the low-risk group was enriched in higher TMB, more abundant immune cells, and subsequently had a brighter prognosis. Except for that, the lower expression of PRGs such as *GZMB*, *IL18*, *IRF1,* and *GZMA* represented better survival, which verified the association between pyroptosis and immune landscape. In conclusion, we performed a comprehensive bioinformatics analysis and established a four-PRG signature consisting of *GZMB*, *IL18*, *IRF1,* and *GZMA*, which could robustly predict the prognosis of BC patients.

## Introduction

Breast cancer (BC) is the most prevalent malignancy and most common cause of cancer-related mortality in women, seriously endangering women’s health and life. The GLOBOCAN2020 reported that BC surpassed lung cancer as the first cause of global cancer incidence in females, with 2,261,419 new cases and nearly 680,000 deaths in 2020 (Hyuna et al., 2021). At present, the common therapeutic methods of BC include surgery, chemotherapy, radiotherapy, and traditional Chinese medicine. With recent advances in medical technology, the diagnosis and treatment of BC is enhanced substantially along with prolonged survival, while long-term survival remains low. In addition, BC is highly heterogeneous and the occurrence and progression are complicated involving multifactorial mechanisms. Therefore, it is essential to carry out an in-depth study of the molecular mechanisms of BC to find the appropriate biomarkers for BC diagnosis and therapy.

Pyroptosis is described as certain programmed cell death induced by inflammasomes and executed via the gasdermin protein, and characterized as releasing inflammatory cytokines, which is involved in various types of cancers, such as colon, liver, and breast (Jianjin et al., 2017). Gasdermin D (*GSDMD*) is a key downstream effector in cell pyroptosis. The average expression level of *GSDMD* in gastric cancer (GC) tissues was lower than that in normal tissues. Knockdown of *GSDMD* could activate JAK/STAT3, PI3K/AKT, and ERK/MAPK pathways, resulting in the tumorigenesis of GC (Jie et al., 2018). Meanwhile, *GSDMD* silencing promoted cell proliferation and mediated malignant biological behaviors through inhibiting PKA signaling pathway (Dan et al., 2019). Similarly, induction of pyroptosis could suppress the proliferative capacity of hepatocellular carcinoma (HC) cells. Studies revealed that NLRP3 expression was significantly lower in tumors compared with normal tissues, which was positively correlated with the histological grade of HC patients (Qing et al., 2014). Moreover, the expression of caspase-1, IL-1β, and *GSDMD* was negative correlation with increasing tumor grade, clinical stage, and poor clinical prognosis in BC, indicating pyroptosis played a central role in BC tumorigenesis and progression (Xia et al., 2020). Based on a close correlation between pyroptosis and cancer progression and prognosis, multiple pyroptosis gene-based studies of prognostic biomarkers have been identified and used for the construction of a gene signature with prognostic predictive power. For instance, seven-gene score, which reflects tumor cell proliferation, is both a prognostic and predictive biomarker for ovarian cancer (Ying et al., 2021). Besides, a “pyroptosis gene regulatory signature” was derived, consisting of nine genes whose expression best predicted skin cutaneous melanoma patient outcome (Anji et al., 2021). However, the prognostic value of pyroptosis gene signature in BC has not yet been fully elucidated.

In summary, we performed a comprehensive analysis of the expression pattern of pyroptosis gene between normal tissues and tumor tissues, and constructed a predictive pyroptosis gene-based risk model, subsequently investigating the correlation between pyroptosis and clinical features, immune microenvironment and immunotherapy responsiveness, providing accurate and efficient diagnostic and prognostic biomarkers of BC.

## Materials and Methods

### Data Collection

The RNA expression profiles, somatic datasets of BC patients, and the corresponding clinical data were obtained using the TCGA database (https://portal.gdc.cancer.gov/), GEO (https://www.ncbi.nlm.nih.gov/geo/), and International Cancer Genome Consortium (ICGC. https://dcc.icgc.org/). Detailed clinical information of the BC patients is displayed in Table 1.

**TABLE 1:** The clinical characteristics of BC patients from the TCGA and GEO databases.

Characteristics		Detailed data		IGGC cohort (*n* = 107)
		TCGA cohort (*n* = 1076)	GEO cohort (*n* = 327)	
Status	Dead	150 (13.94)	103 (25.38)	4 (3.74)
	Survival	926 (86.06)	244 (74.62)	103 (96.26)
Age at diagnosis (years)	≤65	773 (71.84)	305 (93.27)	85 (79.44)
	>65	303 (28.16)	22 (6.73)	22 (20.56)
Gender	Female	1064 (98.88)	327 (100)	107 (100)
	Male	12 (1.12)	—	—
Stage	1	183 (17.01)	—	—
	2	608 (56.51)	—	—
	3	242 (22.49)	—	—
	4	24 (2.23)	—	—
	NA	23 (1.76)	—	—
T	T0		—	3 (2.80)
	T1	281 (26.12)	101 (30.89)	34 (31.78)
	T2	621 (57.71)	188 (57.49)	59 (55.14)
	T3	133 (12.36)	26 (7.95)	6 (5.61)
	T4	38 (3.53)	12 (3.67)	1 (0.93)
	NA	3 (0.28)	—	4 (3.74)
M	M0	895 (83.18)	319 (97.55)	103 (96.26)
	M1	22 (2.04)	8 (2.45)	—
	NA	159 (14.78)	—	4 (3.74)
N	N0	504 (46.84)	137 (41.90)	50 (46.73)
	N1	361 (33.55)	87 (26.61)	37 (34.58)
	N2	120 (11.15)	63 (19.27)	11 (10.28)
	N3	74 (6.88)	40 (12.22)	5 (4.67)
	NA	17 (1.58)	—	4 (3.74)

NA, not available.

### Identification of Differentially Expressed PRGs

A total of 52 PRGs were retrieved from preliminary studies, which are listed in Table 2. To better screen the differentially expressed genes, the “sva” package was performed to normalize the RNA-Seq data to fragment per kilobase million (FPKM) values from the TCGA and GEO datasets to eliminate the batch effect via batch effects correction before comparison. The “limma” package was used to identify differentially expressed genes (DEGs) between BC and normal tissues with the *p*-value < 0.05. Besides we conducted a protein-protein interaction (PPI) network for the DEGs to figure out the interaction via Search Tool for the Retrieval of Interacting Genes (STRING v11.5, https://string-db.org/), in which minimum required interaction score was set as 0.4, representing the degree of the interactions including co-expression, co-occurrence, etc.

**TABLE 2:** 52 pyroptosis-related genes.

Genes	Full-names
AIM2	Absent in melanoma 2
BAK1	BCL2 Antagonist/Killer 1
BAX	BCL2 Associated X
CASP1	Cysteine-aspartic acid protease-1
CASP3	Cysteine-aspartic acid protease-3
CASP4	Cysteine-aspartic acid protease-4
CASP5	Cysteine-aspartic acid protease-5
CASP6	Cysteine-aspartic acid protease-6
CASP8	Cysteine-aspartic acid protease-8
CASP9	Cysteine-aspartic acid protease-9
CHMP2A	Charged multivesicular body protein 2A
CHMP2B	Charged multivesicular body protein 2B
CHMP3	Charged multivesicular body protein 3
CHMP4A	Charged multivesicular body protein 4A
CHMP4B	Charged multivesicular body protein 4B
CHMP4C	Charged multivesicular body protein 4C
CHMP6	Charged multivesicular body protein 6
CHMP7	Charged multivesicular body protein 7
CYCS	Cytochrome C, Somatic
ELANE	Elastase, neutrophil expressed
GPX4	Glutathione peroxidase 4
GSDMA	Gasdermin A
GSDMB	Gasdermin B
GSDMC	Gasdermin C
GSDMD	Gasdermin D
GSDME	Gasdermin E
GZMA	Granzyme A
GZMB	Granzyme B
HMGB1	High mobility group box 1
IL18	Interleukin 18
IL1A	Interleukin 1 alpha
IL1B	Interleukin 1 beta
IL6	Interleukin 6
IRF1	Interferon Regulatory Factor 1
IRF2	Interferon Regulatory Factor 2
NLRC4	NLR family CARD domain containing 4
NLRP1	NLR family pyrin domain containing 1
NLRP2	NLR family pyrin domain containing 2
NLRP3	NLR family pyrin domain containing 3
NLRP6	NLR family pyrin domain containing 6
NLRP7	NLR family pyrin domain containing 7
NOD1	Nucleotide binding oligomerization domain containing 1
NOD2	Nucleotide binding oligomerization domain containing 2
PJVK	Pejvakin/deafness, autosomal recessive 59
PLCG1	Phospholipase C gamma 1
PRKACA	Protein kinase cAMP-activated catalytic subunit alpha
PYCARD	PYD and CARD domain containing
SCAF11	SR-related CTD associated factor 11
TIRAP	TIR domain containing adaptor protein
TNF	Tumor necrosis factor
TP53	Tumor Protein P53
TP63	Tumor Protein P63

### Mutation Analysis of PRGs

The somatic mutation spectrum of 52 PRGs from VARSCAN in BC patients was generated by the “maftools” package based on the MAF in TCGA dataset, which was illustrated in several waterfall plots.

### Consensus Clustering

Consensus clustering is a method that provides quantitative evidence for determining the number and members of possible clusters in a dataset, which uses agglomerative pam clustering with a 1-Pearson correlation distances and resampling 80% of the samples for 10 repetitions, gaining a consensus on an observation’s cluster assignment based on their assignments in all the iterations of the clustering algorithm. R packages “limma” and “ConsensusClusterPlus” were performed to classify the patients based on the DEGs via the suitable clustering variable (k). Besides, R package “survival” and “survminer” were utilized to analyze the correlations between clusters and OS, subsequently presenting the results as Kaplan-Meier (KM) curves.

### Construction and Validation of PRGs-Based BC Prognostic Model

Based on the expression level of the DEGs and the OS of each patient, Cox regression analysis was used to evaluate the correlations between each gene and survival status to select candidate key genes in the TCGA cohort (*p* < 0.05). Meanwhile, the LASSO Cox regression was performed with 10-fold cross-validation and a *p* value of 0.05 for every 1000 cycles to prevent overfitting. SVM (R package “e1071”) and random forest (R package “randomForestSRC”) were then utilized to identify and develop the prognostic model. Ultimately, the risk model was established and calculated by the expression of four genes and their coefficients, which are listed in Table 3. Risk Score = ∑(Xi × Yi) (X: coefficients, Y: gene expression). Based on the median risk score, patients were classified into low- and high-risk groups, and the survival outcomes were investigated between the two subgroups via KM analysis. Besides, principal component analysis (PCA) and t-SNE based on the 4-gene signature were performed via the “t-SNE” R package. The “survival,” “survminer”, and “timeROC” R packages were employed to perform a 1-, 3-, and 5-year ROC curve analysis. For the external validation studies, a BC cohort from the GEO database (GSE20685) was obtained. Following the formula in TCGA, the risk score was calculated and the patients in the GSE20685 cohort were applied to verify the risk model.

**TABLE 3:** The four prognosis-associated PRGs identified by univariate and multivariate Cox regression analysis.

	Univariate Cox analysis				Multivariate Cox analysis	
—	HR	HR.95L	HR.95H	*p*-value		Coefficient
GZMB	0.847	0.744	0.964	0.012	GZMB	−1.318733
IL18	0.759	0.626	0.921	0.005	IL18	−0.543479
IRF1	0.752	0.614	0.922	0.006	IRF1	−0.722430
GZMA	0.825	0.725	0.939	0.004	GZMA	−1.040392

### Independent Prognostic Analysis

The clinical information of patients in the TCGA cohort and the GEO cohort was analyzed in combination with the risk score. We performed univariate and multivariable Cox regression to evaluate whether the risk model was a predictive prognostic factor (Table 4). Moreover, we also developed a nomogram to predict patients’ survival prognosis, which consists of clinical parameters and risk score.

**TABLE 4:** Univariate and multivariate analyses of different clinical characteristics in TCGA cohort and GEO cohort.

TCGA	Univariate Cox analysis				Multivariate Cox regression			
—	HR	HR.95L	HR.95H	*p*-value	HR	HR.95L	HR.95H	*p*-value
Age	1.034	1.019	1.048	<0.001	1.035	1.020	1.050	<0.001
M	6.414	3.604	11.415	<0.001	1.429	0.630	3.240	0.392
N	1.649	1.377	1.975	<0.001	1.187	0.892	1.579	0.239
T	1.570	1.270	1.942	<0.001	1.016	0.756	1.364	0.917
Stage	2.131	1.690	2.687	<0.001	1.616	0.973	2.684	0.064
Risk score	2.691	1.387	5.222	0.003	2.025	1.023	4.009	0.043
**GEO**	**Univariate Cox analysis**				**Multivariate Cox regression**			
—	HR	HR.95L	HR.95H	*p*-value	HR	HR.95L	HR.95H	*p*-value
Age	0.992	0.971	1.014	0.483	1.003	0.983	1.024	0.759
T	1.863	1.440	2.412	<0.001	1.318	0.925	1.880	0.127
N	1.757	1.448	2.134	<0.001	1.665	1.338	2.072	<0.001
M	5.204	2.391	11.326	<0.001	1.367	0.496	3.770	0.546
Risk score	2.713	1.148	6.410	0.023	3.554	1.389	9.092	0.008

HR, hazard ratio.

### Functional Enrichment Analysis

The DEGs between the low- and high-risk groups were filtered according to specific criteria (|log2FC| ≥ 1 and FDR < 0.05) and were carried out with GO and KEGG analyses via the “clusterProfiler” package.

### Immune Infiltration, Tumor Mutation Burden, and Microsatellite-Instability Analysis

We performed the ssGSEA and CIBERSORT to evaluate the correlation between the scores of infiltrating immune cells and the activity of immune-related pathways and the risk score. Besides, Estimation of Stromal and Immune cells in Malignant Tumor tissues using Expression data (ESTIMATE) was conducted to calculate the immune scores which determine stromal score, immune score, estimate score, and tumor purity. In TMB and MSI analysis, Spearman’s correlation analysis was performed to investigate the correlation between TMB and MSI score and gene expression level. Ultimately, we utilized the tumor immune dysfunction and exclusion (TIDE) algorithm to predict potential ICB response of patients in ICGC, which used several gene expression markers to evaluate two different mechanisms of tumor immune escape, including the dysfunction of tumor infiltrating cytotoxic T lymphocytes (CTL) and the rejection of CTL via immunosuppressive factors. The higher the TIDE score, the poorer the efficacy of ICB, the shorter the survival time after ICB treatment.

### Validation of the Expression of the Genes in Risk Signature

All the specimens were from thyroid and breast surgery in Renmin Hospital of Wuhan University. All the patients provided informed consent and were approved by the Ethics Committee of Renmin Hospital of Wuhan University to collect 9 cases of BC tissues and corresponding paracancerous tissues. The clinicopathological parameters of patients are shown in Supplementary Table S1. Total RNA from breast cancer and paracancerous tissue samples was extracted and real-time PCR analysis was performed to validate the expression of the prognostic genes in the risk signature, where GAPDH was used as an internal control.

### Cell Culture

MCF-7 cells were grown in high glucose Dulbecco’s Modified Eagle’s media with 10% fetal bovine serum and 1% penicillin/streptomycin at 37°C and 5% CO_2_. MCF-7 cells were plated on the 6-well plate and grown to 60%. After incubation of different concentrations of LPS (0, 5, 10, 20, 40, and 80 ug/mL), the cell lysate of MCF-7 was extracted and RT-PCR was performed.

### Statistical Analysis

All statistical analyses were conducted with R (v3.6.1). To compare the PRGs expression between the normal and BC tissues and the immune infiltration levels between the high- and low-group, the Wilcoxon test was applied, while the log-rank test was utilized to compare the OS between subgroups.

## Results

### The Potential Association Between Pyroptosis and BC

**FIGURE 1:**
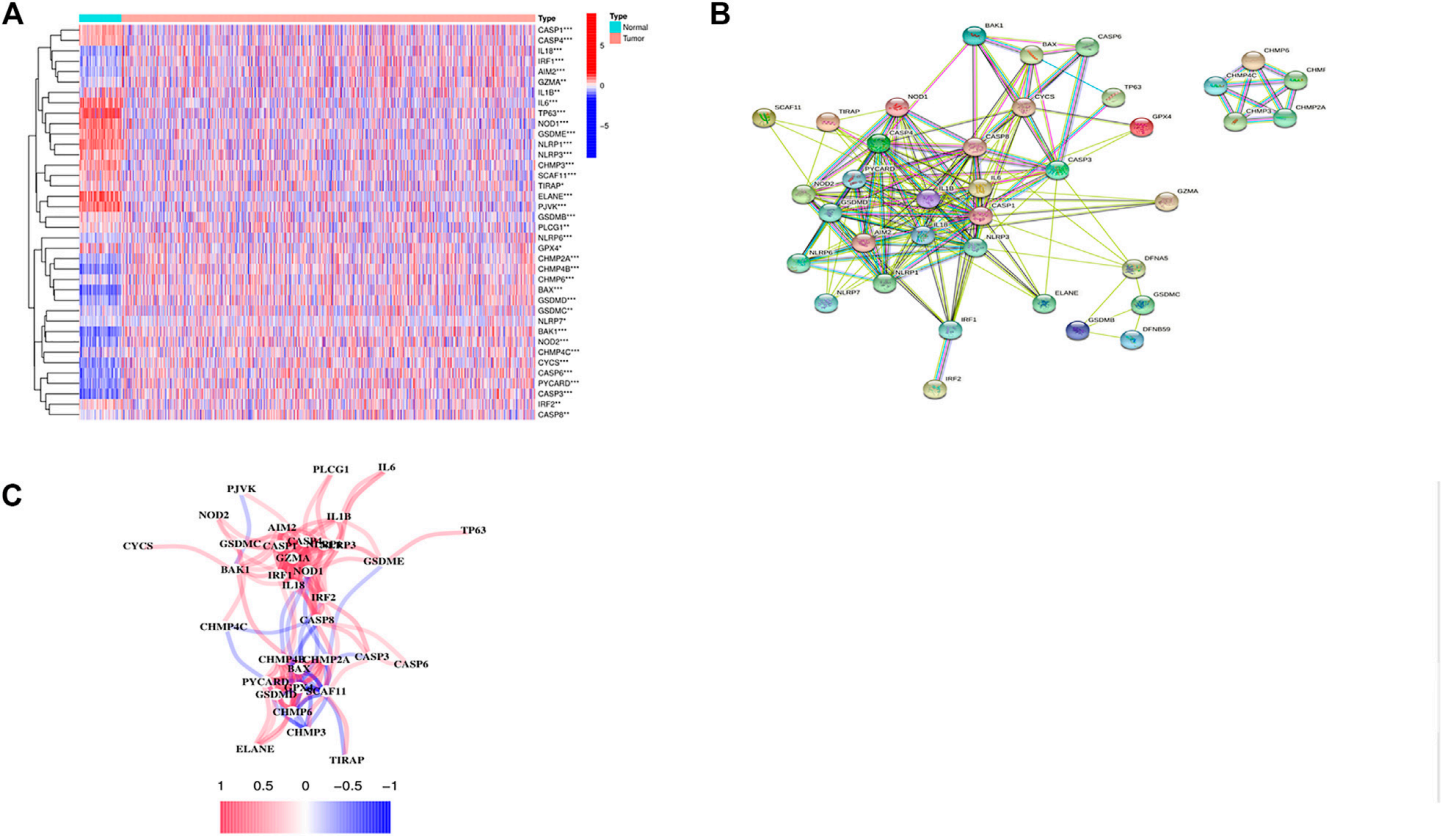
Identification of 38 differentially expressed pyroptosis-related genes and the interactions among them. **(A)** Heatmap of the differential pyroptosis-related gene expression between normal and tumor samples (*: *p* < 0.05, **: *p* < 0.01, ***: *p* < 0.001). **(B)** PPI network showing the interactions of the PRGs (interaction score = 0.4). **(C)** The correlation network of the pyroptosis-related genes.

We compared 52 PRGs expression levels between 113 normal and 1109 tumor tissues from TCGA, and identified 38 DEGs (*p* < 0.05). As shown in Figure 1A, 17 genes (*IL6*, *TP63*, *ELANE*, *NLRP1*, *PJVK*, *GSDME*, *NLRP3*, *NOD1*, *IL1B*, *CASP1*, *CASP4*, *CHMP3*, *SCAF11*, *GPX4*, *IRF2*, *TIRAP*, and *PLCG1*) were downregulated while 21 genes (*CASP8*, *CHMP6*, *GSDMB*, *CHMP4C*, *CHMP2A*, *CHMP2B*, *CYCS*, *CASP3*, *IRF1*, *CASP6*, *BAK1*, *GSDMD*, *GZMA*, *BAX*, *IL18*, *NLRP6*, *NOD2*, *PYCARD*, *AIM2*, *GSDMC,* and *NLRP7*) were upregulated in tumor tissues. PPI networks were constructed to further explore the interactions of these PRGs, which were shown in Figure 1B. The minimum required interaction score for the PPI analysis was set at 0.4, and we determined that *AIM2*, *BAK1*, *BAX*, *CASP1*, *CASP3*, *CASP4*, *CASP8*, *GSDMD*, *IL18,* and *IL6* were hub genes, which had been demonstrated by the correlation network presented in Figure 1C.

### Somatic Alteration of PRGs in BC

**FIGURE 2:**
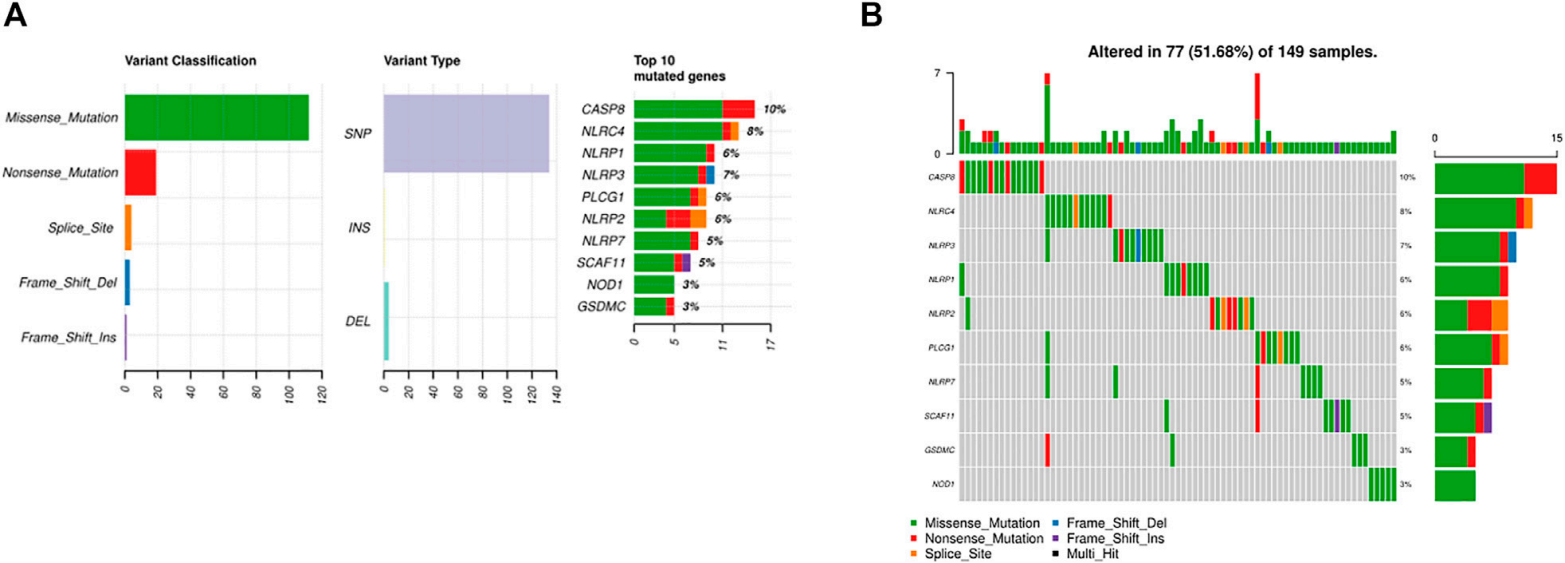
Somatic alterations of PRGs in BC. **(A)** Summary of the mutation in TCGA. **(B)** Waterfall of the mutation in TCGA (the upper pane refers to the frequency of somatic PRG mutations in each patient in the 149 patients; the lower pane refers to the somatic mutation frequency of one PRG in all 149 patients).

As shown in Figures 2A,B, 77 of 149 (51.68%) samples suffered genetic mutations. When referring to variant type, missense mutation was the most frequent variant classification (Figure 2A). In addition, Supplementary Figure S1 illustrated C > T ranked the top in the SNV class. We also demonstrated that *CASP8* was the highest mutated gene accounting for 10%, followed by *NLRC4* and *NLRP3* (Figure 2B).

### Characterization of Different BC Clusters Based on PRGs

**FIGURE 3:**
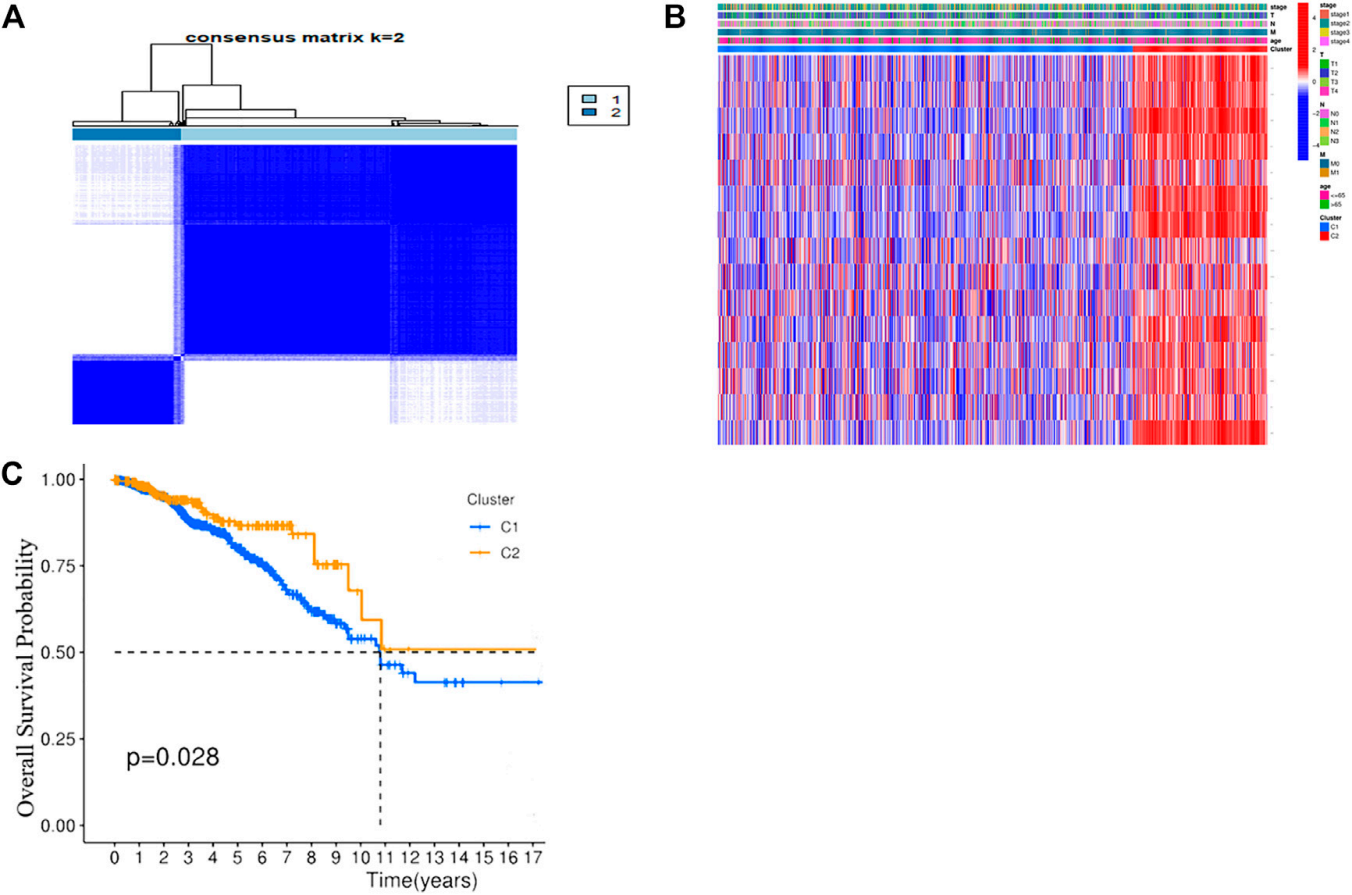
Tumor classification based on the pyroptosis-related DEGs. **(A)** 1076 BC patients were grouped into two clusters according to the consensus clustering matrix (k = 2). **(B)** Heatmap and the clinicopathological characters of the three clusters classified by these DEGs. **(C)** Kaplan–Meier OS curves for the two clusters.

Consensus clustering analysis was performed to explore the correlation between PRGs and BC subtypes in the TCGA cohort. To acquire the most suitable clusters that were characterized as distinct and overlapping, k = 2 were chosen. The results demonstrated that the 1076 BC patients could be well classified into two clusters (Figure 3A) (Supplementary Figure S2). The clinical parameters including the T (T1-4), N(N0-3), M (M0-1), stage (stage1–4), and age (≤65 or >65 years) and the expression profiles of DEGs between the two subgroups (*CASP1*, *CASP4*, *GZMB*, *IL18*, *IL1B*, *IRF1*, *AIM2*, *GSDMB*, *GSDMC*, *IL6*, *NLRC4*, *NLRP1*, *NLRP3*, *TNF,* and *GZMA*) are displayed in a heatmap (Figure 3B). Stepwise, the KM curves showed that cluster 1 had a significantly poorer OS than cluster 2 (*p* = 0.028), suggesting that the PRGs were strongly connected with patients’ survival (Figure 3C).

### Construction of a Pyroptosis-Related Model Through Computational Biology Techniques

**FIGURE 4:**
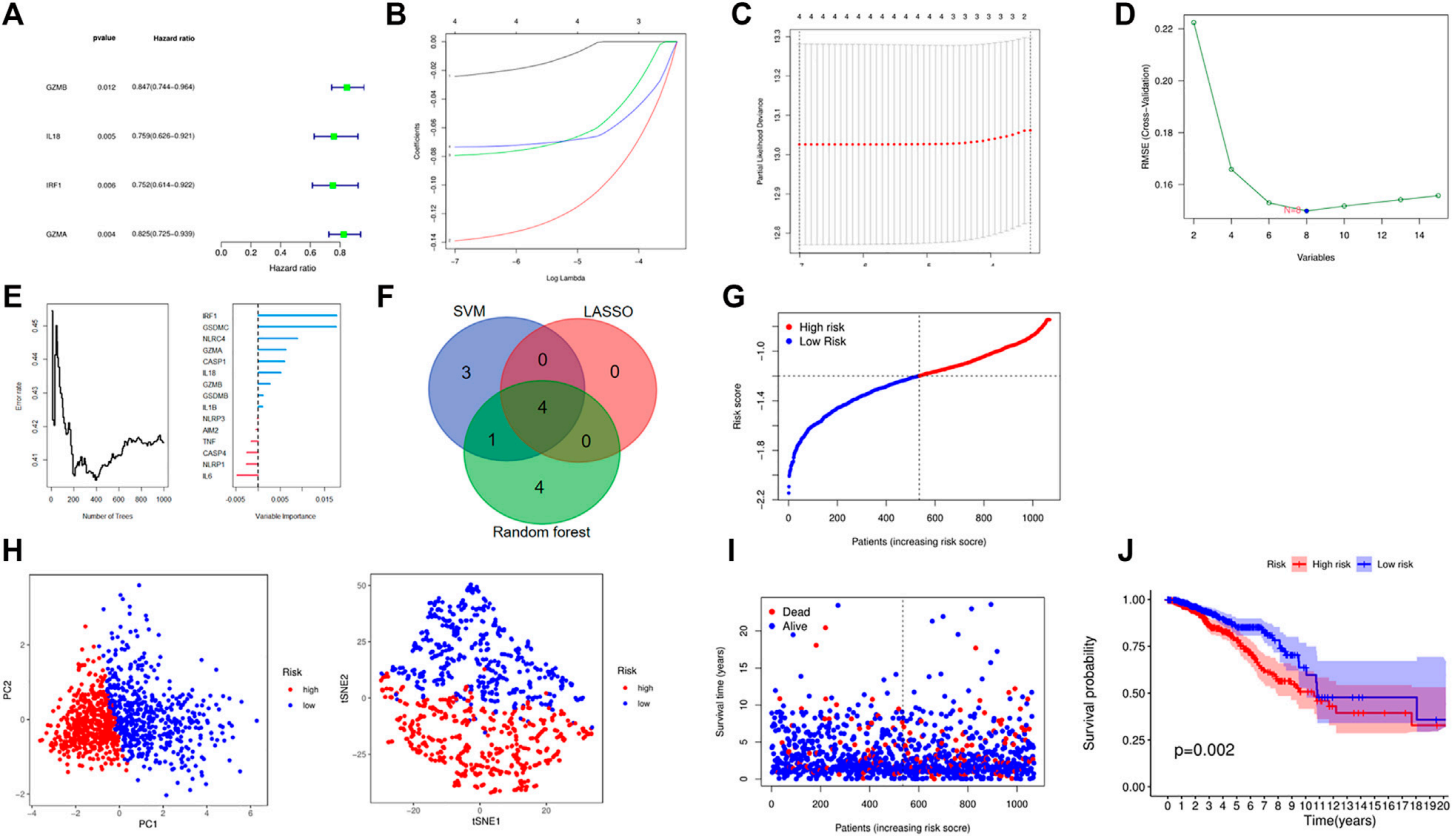
Establishment of a risk model based on TCGA cohort. **(A)** A forest plot consists of four OS-related genes via univariate Cox analysis. **(B–F)** Four OS-related genes signature was constructed through LASSO, SVM, and random forest. **(G)** The distribution and median value of the risk scores. **(H)** PCA and t-NSE plots for patients based on the risk score. **(I)** The distributions of OS status in each patient. **(J)** Kaplan–Meier curves for the OS of patients in the high- and low-risk groups.

**FIGURE 5:**
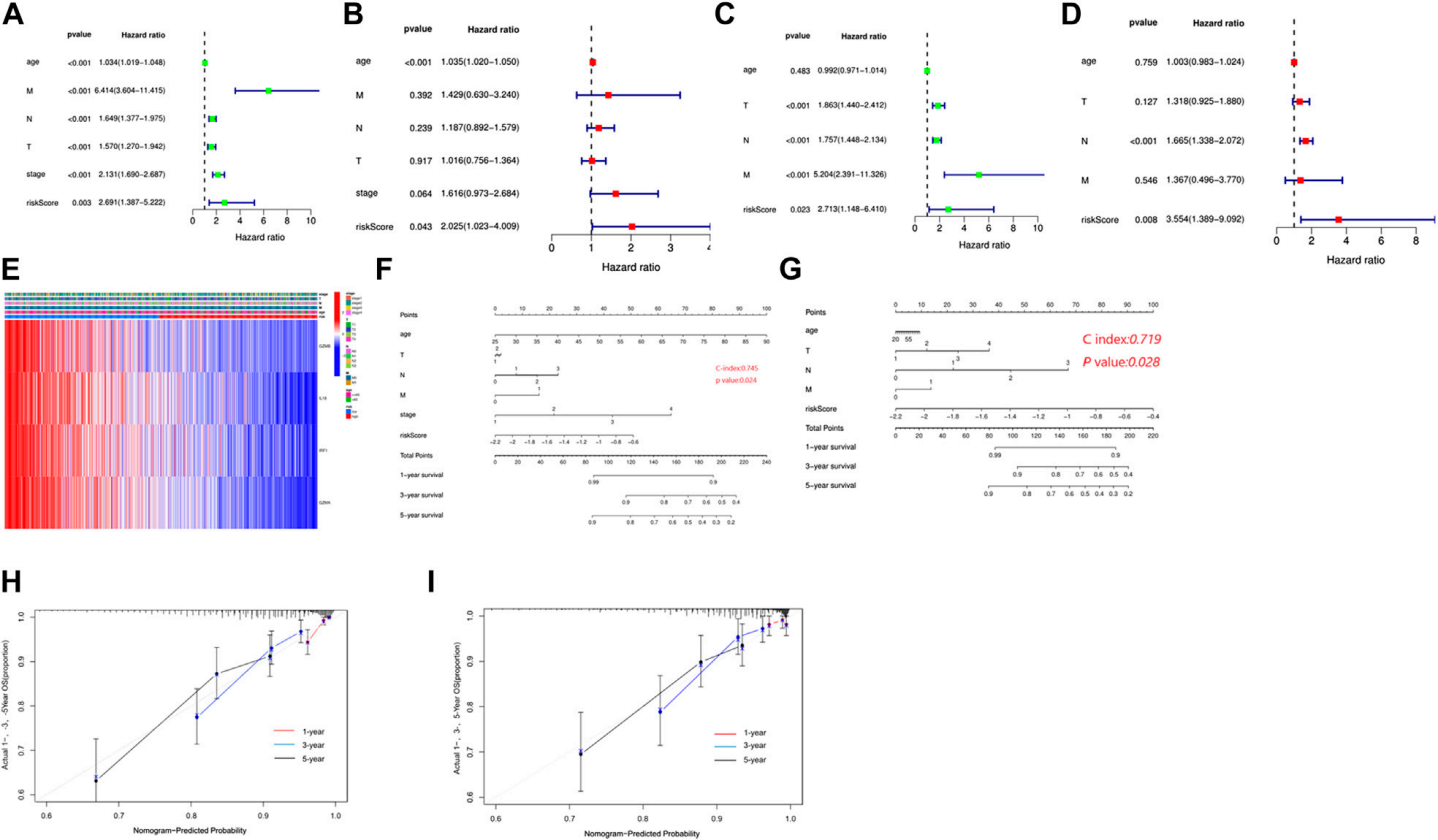
Independent prognostic value of the risk model. **(A)** Univariate analysis in the TCGA cohort. **(B)** Multivariate analysis in the TCGA cohort. **(C)** Univariate analysis in the GEO cohort. **(D)** Multivariate analysis in the GEO cohort. **(E)** Heatmap for the connections between clinicopathological features and the risk groups. **(F)** Construction of a nomogram to predict survival of patients based on clinical parameters and risk score in the TCGA cohort. **(G)** Construction of a nomogram to predict survival of patients based on clinical parameters and risk score in the GEO cohort. **(H)** Calibration curves for the nomogram based on the TCGA cohort. **(I)** Calibration curves for the nomogram based on the GEO cohort.

After integrating the gene expression and clinical information, four candidate prognosis-related PRGs were identified via Cox regression analysis (Figure 4A). Further performing the least absolute shrinkage and selection operator (LASSO) regression analysis, SVM, and random forest, a 4-gene signature was constructed, which consisted of *GZMB*, *IL18*, *IRF1*, and *GZMA* (Figures 4B,C). Eventually, the risk-score equation was as follows: risk score = (−0.024* *GZMB* exp.) + (−0.139* *IL18* exp.) + (−0.079* *IRF1* exp.) + (−0.143* *GZMA* exp.). The risk score of the patients was calculated and ranked according to the above formula, where 1076 patients were divided into low- and high-risk subgroups (Figure 4D). The principal component analysis (PCA) and t-SNE showed that patients with different risks were well separated into two clusters (Figure 4E). High-risk patients showed the significantly lower OS, which was observed via the KM curve (Figures 4F,G). Besides, a total of 327 BC patients from a GEO cohort (GSE28065) were applied to assess the reliability and robustness. Based on the same risk score in the TCGA cohort, 327 patients in the GEO cohort were divided into the high- and low-risk group, respectively (Supplementary Figure S3A). Supplementary Figure S3B shows satisfactory separation between the two subgroups via PCA and t-SNE. Besides, the distribution of risk score and OS of patients was exhibited in Supplementary Figure S3C. What is more important, survival analysis indicated that there was remarkable difference between the low- and high-risk groups, demonstrating good predictive accuracy (*p* =  0.045, Supplementary Figure S3D). Eventually, we compared the predictive value between the risk model and other established signatures, which demonstrated that our risk model had a higher C-index and better prognostic efficiency (Figure 5E **)**.

### Independent Prognostic Value of the Risk Model

To evaluate the independence of the risk model, univariate and multivariable Cox regression analyses were applied. The univariate Cox analysis revealed that age, T stage, N stage, M stage, and risk score were independent factors affecting the prognosis of BC patients, multivariate analysis demonstrated that age and risk score were independent factors in the TCGA cohort (Figure 5A,B). In the GEO cohort, both univariate and multivariate analysis implied that the risk score was a prognostic factor (Figures 5C,D). Meanwhile, we generated a heatmap of clinical features for the TCGA cohort, but there was no significant difference between clinical parameters and survival (Figure 5E). Based on the clinicopathologic features and the risk score, we constructed a predictive nomogram to predict the survival probability. The predictive nomogram was applied to predict 1-year, 3-year, and 5-year survival probabilities of BC patients in TCGA and GEO (Figures 5F,G). Furthermore, the calibration and the C-index confirmed the promising ability to predict the survival of patients (Figures 5H,I).

### The Risk Model Implicated Strong Association With Landscape of Immune Microenvironment

**FIGURE 6:**
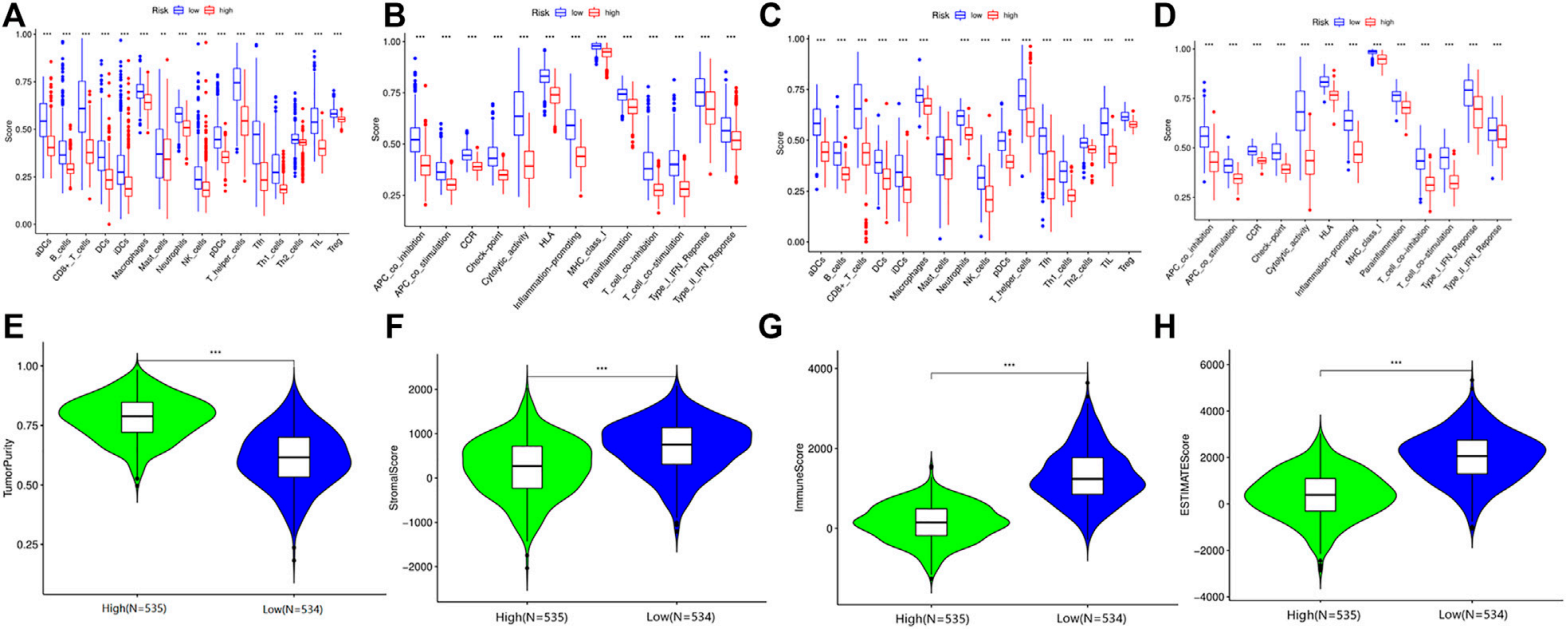
Immune microenvironment between high- and low-risk group patients in the TCGA and GEO cohorts. **(A)** Comparison of the enrichment scores of 16 types of immune cells between high-risk and low-risk groups in TCGA. **(B)** Comparison of the enrichment scores of 13 immune-related pathways between high-risk and low-risk groups in TCGA. **(C)** Comparison of the enrichment scores of 16 types of immune cells between high-risk and low-risk groups in GEO. **(D)** Comparison of the enrichment scores of 13 immune-related pathways between high-risk and low-risk groups in GEO. **(E)** The relationship between tumor purity and risk score in the TCGA cohort. **(F)** The relationship between stromal score and risk score in the TCGA cohort. **(G)** The relationship between immune score and risk score in the TCGA cohort. **(H)** The relationship between estimate score and risk score in the TCGA cohort.

To further understand the biological function of the DEGs between the high- and low-risk groups based on the prognostic risk model, we identified 179 DEGs via the “limma” package (FDR<0.05). Of these DEGs, 177 genes were downregulated, while another two genes were upregulated in the high-risk group (Table 5). Gene ontology (GO) enrichment analysis and Kyoto Encyclopedia of Genes and Genomes (KEGG) pathway analysis were then employed, demonstrating that the DEGs were mainly associated with the regulation of lymphocyte activation, external side of plasma membrane, antigen binding, and tryptophan metabolism (Supplementary Figure S4). According to the functional analyses, it was interesting to observe that the biological processes were enriched in immune functions and pathways, indicating the risk model was closely associated with immune responses. Therefore, we further conducted and compared the enrichment scores of 16 types of immune cells and the activity of 13 immune-related pathways between the low- and high-risk groups via the single-sample gene set enrichment analysis (ssGSEA). Compared with the high-risk group, the low-risk subgroup generally had higher levels of infiltration of immune cells, which included aDCs, B cells, CD8^+^ T cells, DCs, iDCs, macrophages, mast cells, neutrophils, natural killer (NK) cells, T helper (Th) cells, tumor-infiltrating lymphocytes (TILs), and regulatory T (Treg) cells in TCGA cohort (Figure 6A). Additionally, the low-risk group also exhibited higher activity in immune pathways than in the low-risk group in the TCGA cohort (Figure 6B). Similar conclusions were presented when retrieving the immune status in the GEO cohort (Figure 6C). Except for the mast cells, the other immune cells were lower in the high-risk group (Figure 6D). Furthermore, the R package “estimate” was utilized to investigate the relativity between tumor microenvironment and risk score in TCGA. These results confirmed that the risk score was positively correlated with tumor purity, while negatively with stromal score, immune score, and estimate score (Figures 6E–H).

**TABLE 5:** DEGs between the low- and high-risk groups in the TCGA cohort.

Gene	Low mean	High mean	logFC	FDR
CORO1A	5.360104747	4.039533552	−1.320571195	1.4533E-102
ICOS	2.66870652	1.401707634	−1.266998885	6.0567E-99
ADAMDEC1	4.067634374	2.677191427	−1.390442946	1.58179E-61
UBASH3A	2.396795426	1.235868978	−1.160926447	4.8647E-113
SLPI	6.417742723	5.374261184	−1.043481539	2.82123E-15
IL2RG	5.381610354	3.590786963	−1.790823392	6.3889E-123
LTB	4.162616237	2.693823165	−1.468793072	1.12041E-83
KLHDC7B	3.641745108	2.602829947	−1.03891516	4.84572E-44
CD6	2.907802118	1.86808555	−1.039716568	2.6307E-108
GZMA	4.924957952	3.098356833	−1.826601119	6.4323E-129
CD247	3.313011386	2.175286145	−1.137725241	2.4537E-114
GBP4	4.608669232	3.234306381	−1.374362851	8.51808E-87
CYBB	4.794343167	3.677356315	−1.116986852	2.25184E-71
ZAP70	3.006312166	1.979412401	−1.026899765	4.3563E-100
LGALS9	5.115470076	4.114339016	−1.00113106	4.46913E-83
CCL18	3.924514642	2.60205151	−1.322463132	2.78856E-41
SLAMF8	4.366530125	3.086536589	−1.279993536	5.46533E-90
EEF1A2	4.563524061	5.738800508	1.175276447	7.28811E-16
GBP1P1	2.449571975	1.27318215	−1.176389825	2.69048E-88
IDO1	4.011478175	2.390257247	−1.621220928	1.22696E-94
APOE	8.128534974	7.095641377	−1.032893598	3.36347E-46
SPI1	4.725939757	3.587575922	−1.138363835	1.42452E-90
CPB1	2.939800849	4.193458095	1.253657245	3.63074E-08
CCL4	4.115440664	3.03068862	−1.084752045	3.2697E-93
C1S	7.307363861	6.222951045	−1.084412816	1.03439E-52
PTGDS	4.923081328	3.531880381	−1.391200948	1.16044E-55
RAC2	5.72871771	4.525700365	−1.203017345	3.91066E-91
IGHD	3.051991654	2.02802661	−1.023965043	4.26743E-34
BIN2	3.709266834	2.559470948	−1.149795886	2.8028E-109
CCR5	4.284251105	2.993071484	−1.291179621	1.0578E-116
GPR171	2.881081182	1.79534601	−1.085735173	6.2069E-100
S1PR4	2.787226885	1.7424426	−1.044784285	3.5729E-100
AOAH	3.513732239	2.494757235	−1.018975004	3.01337E-95
CST7	4.595045167	3.165546127	−1.42949904	8.5695E-104
HLA-DPB1	7.642577226	6.371570292	−1.271006934	9.7664E-101
TNFRSF17	2.95989884	1.840601865	−1.119296975	2.05885E-53
HCP5	5.290979401	4.240242684	−1.050736717	6.1271E-58
CD3E	4.527286876	2.861578069	−1.665708807	5.2271E-116
SPOCK2	4.154981604	2.896084803	−1.258896802	4.43455E-95
SELPLG	5.040260081	3.923229581	−1.1170305	5.03095E-93
NKG7	4.733281588	2.848564769	−1.884716819	2.8775E-122
S100A8	4.508000675	3.100904178	−1.407096498	3.4299E-31
LTF	7.30144272	5.734845084	−1.566597636	6.46878E-19
CARD16	3.450089988	2.437280475	−1.012809513	1.9148E-110
IGLV1-44	6.246509235	4.17187417	−2.074635065	1.66624E-49
IGHG1	7.350130441	4.929048971	−2.42108147	3.76055E-51
S100A9	6.939100023	5.333109776	−1.605990247	7.49594E-29
GZMB	3.70949304	2.070526724	−1.638966316	1.5122E-101
HLA-DRA	10.56132681	9.157501678	−1.403825133	7.2754E-108
S100A7	4.069054754	2.809805452	−1.259249301	1.11916E-12
CD79A	4.366519802	2.713664675	−1.652855127	6.25078E-67
FDCSP	4.642483781	2.985719615	−1.656764166	1.28725E-27
IGHV1-69	3.315959777	1.963011155	−1.352948622	3.55083E-37
HLA-DPA1	7.39906434	6.063546257	−1.335518083	1.13415E-93
TNFRSF1B	5.181175125	4.097575956	−1.083599169	7.1194E-103
TRAC	5.783767342	4.117421753	−1.666345589	1.2437E-105
ACKR1	4.484716745	3.392594695	−1.09212205	8.43227E-23
SELL	4.700846275	3.272053084	−1.428793192	1.31627E-82
GZMM	3.05536066	1.892536439	−1.162824222	7.4963E-92
LCK	3.944866286	2.52912373	−1.415742556	1.7523E-110
HLA-DQA1	6.247503546	4.076673578	−2.170829968	3.76188E-89
CXCL10	6.975795807	4.976685426	−1.999110381	1.686E-81
SIT1	3.28843416	1.882746759	−1.405687401	9.2293E-112
PRF1	3.668183558	2.442155392	−1.226028166	1.4193E-115
C1QC	7.845681197	6.675610114	−1.170071082	1.0383E-82
LAG3	3.106890931	2.086277781	−1.020613151	1.79066E-80
CCL2	5.46286324	4.337065426	−1.125797814	4.88778E-61
APOL1	6.050047347	4.908171095	−1.141876252	3.70502E-84
IL18	4.190789992	3.044475659	−1.146314333	9.6008E-126
HLA-F	6.106118877	4.956620172	−1.149498705	1.21661E-79
CTSW	3.397907205	1.869805587	−1.528101618	5.75927E-97
RARRES1	5.269708166	3.950770119	−1.318938047	5.39529E-42
IGKC	8.658650476	6.398117726	−2.26053275	1.0066E-54
LAMP3	3.99196859	2.679670854	−1.312297736	1.17868E-77
BCL2A1	3.920467154	2.715766402	−1.204700753	1.7772E-80
CD79B	3.002966686	2.001833894	−1.001132793	7.4998E-68
FERMT3	4.761586406	3.581359542	−1.180226864	1.5344E-99
IGHM	7.383651483	5.072475929	−2.311175554	6.47352E-53
IL2RB	4.231582771	3.018756424	−1.212826348	1.42754E-92
CXCL9	6.821303571	4.359150742	−2.462152829	2.06771E-90
C1QB	7.515156442	6.219815826	−1.295340615	4.79414E-89
NAPSB	4.233310224	3.091702908	−1.141607316	9.82202E-68
SIRPG	2.47922886	1.374519633	−1.104709228	1.4276E-113
IL32	5.59450272	4.310250032	−1.284252687	4.138E-78
SASH3	4.761827311	3.529669244	−1.232158067	4.8384E-116
TIGIT	3.018274307	1.869993222	−1.148281086	6.3794E-108
MS4A1	3.090610318	1.892911125	−1.197699193	5.40303E-69
CASP1	4.613143497	3.599216242	−1.013927256	1.9168E-100
CD7	3.141448175	1.968091019	−1.173357157	9.1813E-106
CXCR6	2.827605632	1.795738331	−1.031867301	3.9044E-108
HCST	4.46600762	3.295520384	−1.170487236	1.52549E-96
EBI3	3.018209859	1.925793352	−1.092416507	3.48739E-86
IL7R	4.438791573	3.031530025	−1.407261549	8.37936E-83
SLAMF7	3.839990058	2.377133193	−1.462856865	4.71716E-96
IRF8	4.377807761	3.327696457	−1.050111305	4.67308E-92
HLA-DQB1	5.839998886	4.356166276	−1.483832611	7.66425E-80
HLA-DMB	5.547993757	4.360576097	−1.18741766	2.0215E-108
EVI2B	5.153593436	3.938844092	−1.214749344	1.39392E-98
SPN	2.631629192	1.615489013	−1.016140178	1.73311E-97
HLA-DMA	6.717204277	5.498473069	−1.218731209	4.0656E-113
TAP1	6.638832453	5.48554722	−1.153285233	5.03798E-65
PLA2G2D	2.400055862	0.99788987	−1.402165992	6.14283E-83
CYTIP	4.182375059	3.049161213	−1.133213846	2.2841E-87
CXCL13	5.554707054	3.423637016	−2.131070037	1.90005E-64
CLEC10A	3.141723147	2.071571778	−1.070151369	4.2604E-67
WAS	4.161853167	3.138125438	−1.023727729	2.2478E-113
HLA-DQB2	4.483645366	3.346969498	−1.136675869	5.5854E-58
CD3G	2.782069722	1.633347888	−1.148721835	3.0213E-106
IL10RA	4.492164569	3.373913704	−1.118250865	2.8396E-102
HCLS1	5.365722359	4.240379245	−1.125343114	3.8519E-104
CCL21	4.486810762	3.239732148	−1.247078614	1.88152E-29
FPR3	4.942062247	3.926922957	−1.015139291	1.50175E-51
SRGN	7.136395383	5.906759073	−1.22963631	1.31748E-95
GPR183	4.741687685	3.523698318	−1.217989367	2.08636E-75
MAP4K1	3.200923701	2.039054283	−1.161869419	1.046E-102
CD5	3.367912498	2.097936429	−1.269976068	4.6643E-107
CD53	5.610501937	4.295049786	−1.315452152	3.241E-111
HLA-DOA	4.210149047	3.164637444	−1.045511603	1.03338E-83
CD3D	4.941889641	3.141417665	−1.800471975	6.9829E-123
GNLY	3.208806672	1.934835347	−1.273971325	7.82857E-97
GBP5	3.888090151	2.392565378	−1.495524773	5.19835E-98
CXCL11	4.77682554	3.035835962	−1.740989578	1.85212E-78
BIRC3	4.341237451	3.190572681	−1.150664769	2.20649E-78
C16orf54	3.415507471	2.285109419	−1.130398052	2.473E-103
CD69	3.663594833	2.634742101	−1.028852731	4.16642E-80
C1QA	7.688633915	6.330589492	−1.358044423	2.98309E-92
DOK2	3.688886573	2.617313807	−1.071572767	1.35046E-95
CD48	4.344626171	2.991797311	−1.35282886	1.8574E-117
FGL2	5.113359749	3.994426038	−1.118933711	1.55171E-76
TRAV12-2	2.127961852	1.03905828	−1.088903573	2.2292E-93
GIMAP4	5.464517511	4.410288234	−1.054229277	1.84947E-93
GBP1	5.840635431	4.424450267	−1.416185164	5.68627E-84
TRAT1	2.500318227	1.450122923	−1.050195304	3.3268E-104
C3	7.838554587	6.53817962	−1.300374968	1.50441E-66
CD37	3.979895154	2.860805383	−1.119089771	5.0979E-105
IGLL5	5.626926768	3.830681897	−1.796244871	2.14691E-51
IGLV6-57	4.440534612	2.603583196	−1.836951416	1.35126E-44
CCL8	4.013560591	2.961382408	−1.052178183	1.42265E-51
TYROBP	7.265365441	6.236943531	−1.028421911	3.18142E-82
FCER1G	6.731394543	5.710139026	−1.021255517	3.01917E-84
CD8A	4.139211436	2.692320927	−1.44689051	3.1195E-104
KLRB1	3.224712682	2.122700348	−1.102012334	6.13308E-89
CD27	4.109424689	2.714609904	−1.394814785	2.27118E-97
MMP7	5.712559584	4.574873337	−1.137686247	1.43786E-22
CD52	6.214804266	4.389171799	−1.825632467	2.4335E-105
APOC1	7.08329022	6.015604239	−1.067685981	6.72133E-52
GIMAP7	5.057493352	3.934627555	−1.122865798	2.75875E-78
CXCR3	3.322254993	1.927552465	−1.394702528	7.9032E-116
PLEK	4.988885801	3.71449876	−1.274387041	2.661E-99
CCR7	3.766279493	2.452475926	−1.313803567	2.67691E-90
GMFG	5.171020452	4.120109114	−1.050911339	4.5574E-101
CD2	5.421111687	3.538445918	−1.882665769	1.8373E-123
CD96	2.855881742	1.696207766	−1.159673976	5.8805E-111
TMC8	3.47037334	2.442798459	−1.027574881	1.1318E-108
HLA-B	10.5049036	9.455108846	−1.049794758	2.70117E-74
LYZ	7.517179112	5.747192503	−1.76998661	5.72105E-71
SLAMF6	3.41444143	2.202687773	−1.211753656	4.5438E-107
CHI3L1	5.21416871	4.140128252	−1.074040459	5.67107E-26
LGALS2	3.404502369	2.038701675	−1.365800694	1.60395E-76
ITGB2	5.286587731	4.082371297	−1.204216433	1.02151E-83
PTPRC	4.462383664	3.114560617	−1.347823047	2.42856E-89
GZMK	4.219947755	2.510364693	−1.709583061	3.1821E-105
CSF2RB	4.130809361	3.110874429	−1.019934932	4.88547E-79
IL4I1	3.931211922	2.677897202	−1.25331472	8.04983E-70
GZMH	3.385255319	1.994160798	−1.391094521	5.0674E-107
CCL19	5.997614042	3.970147578	−2.027466464	8.17781E-60
HLA-DOB	3.132584238	2.056886162	−1.075698075	2.7306E-84
SH2D1A	2.744864409	1.567963027	−1.176901382	4.9839E-110
CD38	2.881080346	1.829525409	−1.051554937	2.93629E-86
MPEG1	5.038123971	3.940072182	−1.098051789	1.1602E-76
CD4	4.726462006	3.613613486	−1.11284852	1.2146E-102
HLA-DRB6	5.062636453	3.814124126	−1.248512327	2.05324E-71
PSMB9	5.736704054	4.411715021	−1.324989032	1.83162E-95
IRF1	5.078369975	4.069631847	−1.008738129	1.8158E-107
CD74	9.735425117	8.383650479	−1.351774638	3.9705E-115
LAPTM5	7.783373879	6.703807082	−1.079566797	4.65831E-94
CTSS	6.315416511	5.053186817	−1.262229694	2.2885E-96
AIF1	5.469002536	4.38958142	−1.079421115	2.505E-103
CCL5	6.565983508	4.546408762	−2.019574746	3.34E-120

### Low-Risk Patients Predicted More Sensitive Immunotherapies and Favorable Prognosis

**FIGURE 7:**
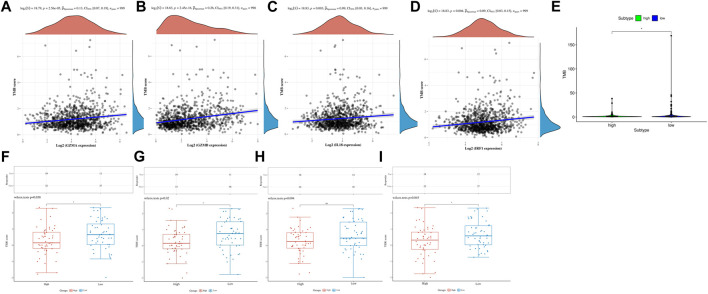
TMB, MSI, and ICB analysis of PRG in the TCGA and ICGC cohorts. **(A)** The correlation between GZMA and TMB in TCGA. **(B)** The correlation between GZMB and TMB in TCGA. **(C)** The correlation between IL18 and TMB in TCGA. **(D)** The correlation between IRF1 and TMB in TCGA. **(E)** The correlation between risk score and TMB in TCGA. **(F)** The distribution of immune response scores between high and low expression of GZMA in ICGC. **(G)** The distribution of immune response scores between high and low expression of GZMB in ICGC. **(H)** The distribution of immune response scores between high and low expression of IL18 in ICGC. **(I)** The distribution of immune response scores between high and low expression of IRF1 in ICGC.

Several studies have shown that patients with higher TMB were associated with enhanced response, long-term survival, and long-lasting clinical benefits when receiving immune checkpoint blocking therapy (Bo et al., 2020). MSI was also identified as a predictive biomarker for cancer immunotherapy (Liisa et al., 2018). Pearson analyses were conducted to investigate the correlation between PRGs and TMB, MSI, and ICB in BC, thus investigating whether these PRGs could also serve as biomarkers for immunotherapy. The results revealed a positive correlation between TMB and *GZMA* (Figure 7A, *p* = 2.56e-5), *GZMB* (Figure 7B, *p* = 2.45e−16), *IL18* (Figure 7C, *p* = 0.003), and *IRF1* (Figure 7D, *p* = 0.004). More importantly, we also deeply explore the relationship between TMB and risk in TCGA cohort, indicating that patients in the high-risk group had a poorer TMB than the low-risk group (Figure 7E *p* < 0.05). However, there was no significant correlation between MSI and *GZMA*, *GZMB*, *IL18*, *IRF1*, and risk score in MSI analysis (Supplementary Figure S5). TIDE was used to evaluate two different mechanisms of tumor immune escape, thus providing predicted results about immunotherapy (Peng et al., 2018). The higher the TIDE score, the worse the efficacy of ICB, subsequently the shorter the OS. To better illustrate the predictive power of the PRGs for immunotherapy, TIDE was applied in the ICGC cohort. Surprisingly, the results indicated that the expression of *GZMA*, *GZMB*, *IL18*, and *IRF1* were negatively correlated with TIDE and positively correlated with ICB (Figures 7F–I).

### The Distinction of the Immune Status and Immunotherapy Between the Two Groups

**FIGURE 8:**
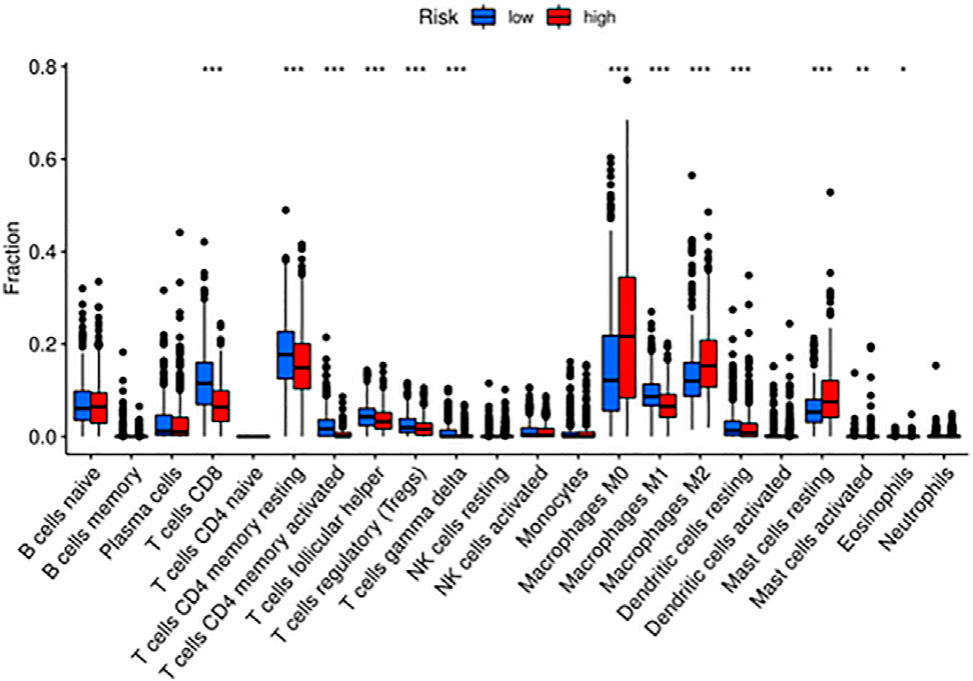
The enriched immune cells and immune functions between high- and low-risk groups via CIBERSORT.

**FIGURE 9:**
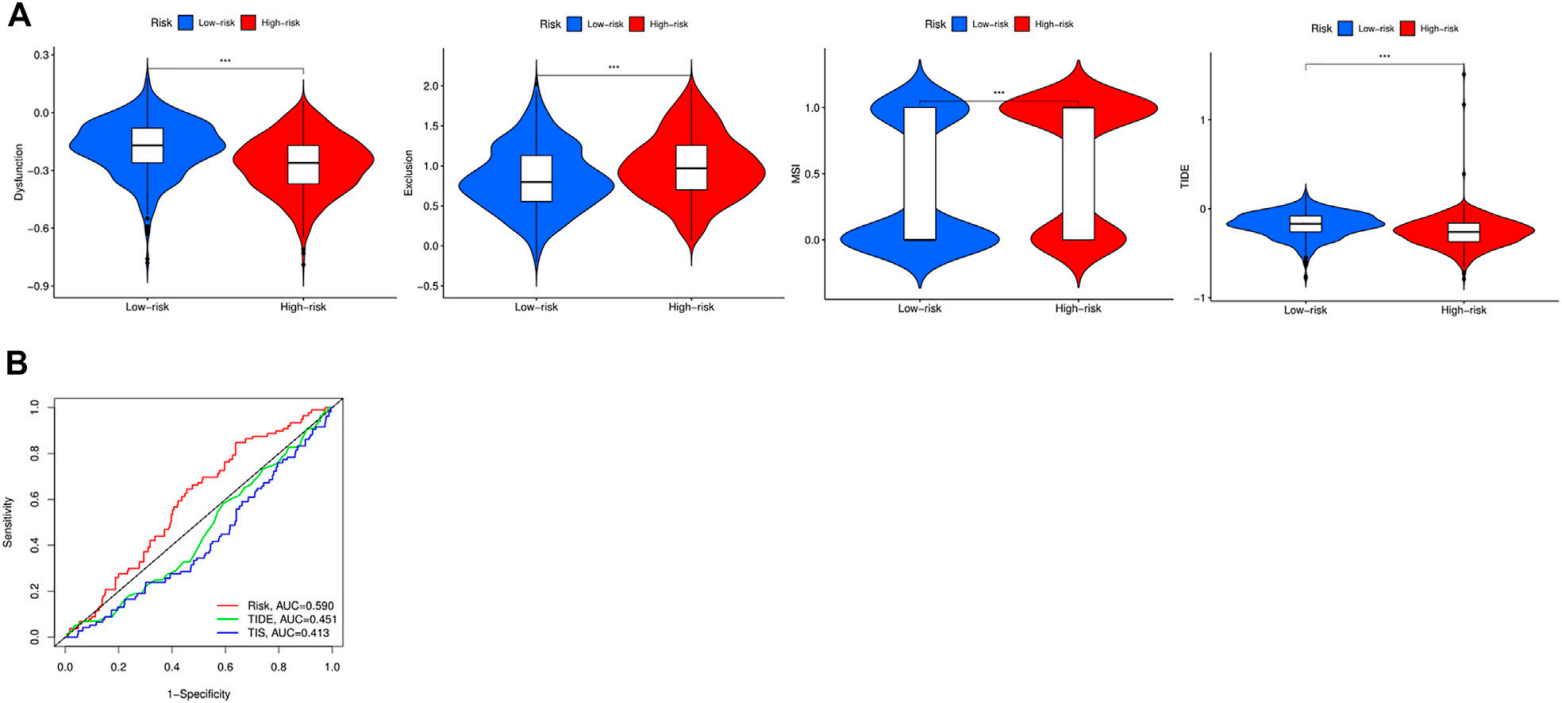
Evaluation and comparison of the TIDE score between high- and low-risk groups. **(A)** The comparisons of the dysfunction, exclusion, MSI, and TIDE scores between high- and low-risk groups. **(B)** The comparison of the predictive efficiency between the model and TIDE, TIS scores.

To further investigate the relationship between the immune status and the risk groups, GSEA was employed to demonstrate different biological functions and pathways between groups (Supplementary Figure S6A). The KEGG results indicated that “natural killer cell mediated cytotoxicity” pathway was enriched in the low-risk group. Besides, “adaptive immune response” was found to be enriched in the low-risk group, which exhibited certain associations between immunity and risk (Supplementary Figure S6B). Meanwhile, we compared the enrichment of 22 kinds of immune cells between high- and low-risk groups, which showed that the low-risk group generally had higher levels of immune cells, such as CD8^+^T cell, CD4^+^T cell, T helper cell, regulatory T cell, gamma delta T cell, M1 macrophage, resting dendritic cell, and lower levels of M0, M2 macrophages and resting mast cells, than the high-risk group (Figure 8). Not only that, but also the Kaplan-Meier curves with immune cells and immune functions are listed in Supplementary Figures S7 and S8. To illustrate the relationship between immunotherapeutic responses and the risk, we explored the expression of several immune checkpoints. As shown in Supplementary Figure S9, all immune checkpoints were overexpressed in the low-risk group, such as *CD274*, *CTLA4*, *HAVCR2*, *LAG3*, *PDCD1*, *PDCD1LG2*, *TIGIT,* and *SIGLEC15*, indicating that patients in the low-risk group were more susceptible to immunotherapy. Then TIDE was utilized to assess the immunotherapy response of patients in the two groups. TIDE, incorporating various gene expression markers to evaluate tumor immune escape mechanisms, could predict potential immunotherapy response. The higher the TIDE score, the worse the efficiency of the immune checkpoint blocking therapy, subsequently the shorter the survival. In our results, the high-risk group had a lower TIDE score, representing that high-risk group patients responded to immunotherapy more effectively. Also, the results indicated that the high-risk group had a higher MSI and T cell exclusion score, while the low-risk group had a higher T cell dysfunction score. Eventually, we compared the prognostic values of the risk model, TIDE, and T-cell-inflamed signature (TIS) (Figure 9A). Interestingly, our risk model might have better sensitivity and specificity than they do (Figure 9B).

### The Expression Validation of the Candidate Genes

**FIGURE 10:**
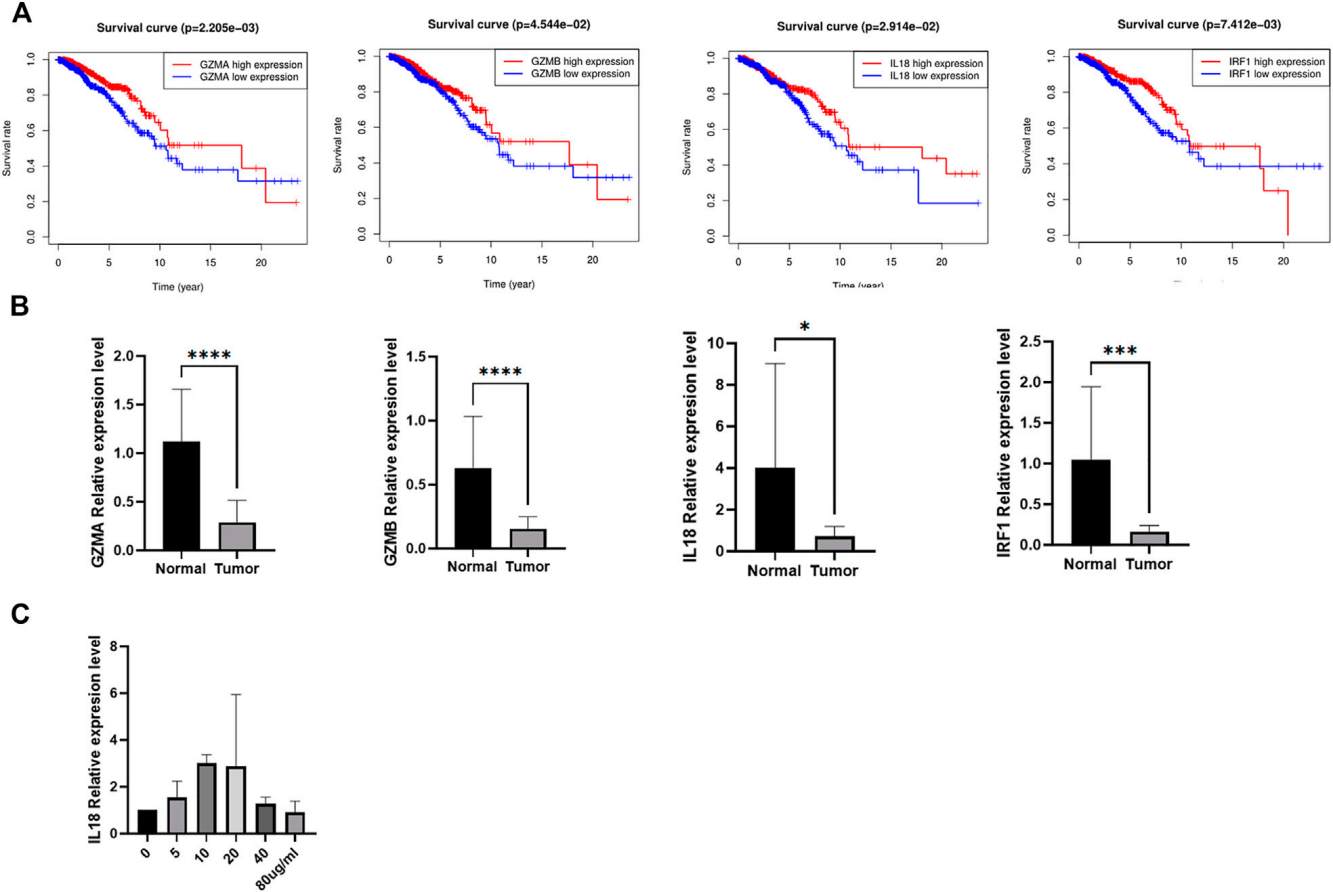
The expression validation of the candidate genes. **(A)** The Kaplan-Meier curves of BC patients between high and low expression of the candidate genes. **(B)** The expression of the four candidate genes between tumor and normal tissues. **(C)** The mRNA expression of IL 18 after incubating via different concentrations of LPS in MCF-7.

We investigated the prognostic values of the four genes, higher expression of *GZMA*, *GZMB*, *IL18*, and *IRF1* indicated better prognosis (Figure 10A). To further confirm the expression of the four prognostic genes, we obtained BC and normal breast tissues from patients including 9 Lumina A cancer samples and 9 normal tissues. Consistent with our results in the database, *GZMA*, *GZMB*, *IL18*, and *IRF1* were decreased in tumor tissues than normal tissues (Figure 10B). In addition, to further investigate the role of IL18 in BC cells, we activated the NF-kB pathway via lipopolysaccharide (LPS) in MCF-7 and then measured the expression of IL18 in those cells using RT-PCR. The results indicated that the expression of IL18 was significantly increased when treated with different concentrations of LPS (Figure 10C). Besides, IL18 was found to be increased in BC samples than normal samples. In general, the above results demonstrated a close relationship between inflammation, pyroptosis, and BC , and further studies were needed to elucidate the mechanism.

## Discussion

Pyroptosis, a form of programmed cell death, has a dual role in tumorigenesis. On the one hand, normal cells go through multiple inflammatory molecules released during pyroptosis, which ultimately lead to their transformation into neoplastic cells (Karki and Kanneganti, 2019). On the other hand, pyroptosis stimulates the accidence of tumor cell deaths, making pyroptosis a promising therapeutic target (Ruan et al., 2020). Hence, it is essential to explore and establish a pyroptosis-related diagnostic and prognostic signature to clarify the significance of pyroptosis. Nevertheless, the specific risk model has not been constructed, which will be accomplished in our study.

In current study, we found that most PRGs are differentially expressed in BC, suggesting the potential role in tumorigenesis, which was consistent with the research by Zhang, that GSDME, functioning as a tumor suppressor, suppressed tumor progression via inducing pyroptosis (Ziwen et al., 2021). Subsequently, we classified BC patients into two different groups based on the expression of PRGs. More importantly, there existed a difference between groups, further demonstrating the possibility of PRGs as prognostic biomarkers.

Next, we performed LASSO Cox regression analysis to construct a prognostic gene model based on four prognostic PRGs (*GZMB*, *IL18*, *IRF1*, and *GZMA*), which could predict the overall survival of BC patients.

Granzyme B (*GZMB*), a main member of a family of serine proteases, is also a toxic granule secretase produced by cytotoxic T lymphocytes and natural killer cells in the tumor microenvironment. In addition, *GZMB* could cleave and thereby activate downstream caspase-3 and promote apoptosis of target cells owing to the hydrolytic activity. Similar with the above site, *GZMB* cleaved gasdermin E (*GSDME*) to cause pyroptosis instead of apoptosis, thus enhancing the patients’ anti-tumor immune response, which inhibits tumor growth (Zhibin et al., 2020). A recent study found that *GZMB* was highly expressed in higher infiltrating T lymphocytes, while downregulated in CRC with vascular invasion, lymphatic invasion, and lymph node positivity, indicating that the downregulation of *GZMB* strongly correlated with early metastasis in CRC (Paul et al., 2011). Meanwhile, the prognostic gene model of CRC found that the higher the expression level of *GZMB*, the longer the OS of patients, suggesting that *GZMB* functioned as a promising tumor suppressor in CRC. Enrichment analysis also demonstrated that *GZMB* participated in the occurrence and development of CRC via regulating immune-related signaling pathways (Yuanyu et al., 2020). Intensive studies have been conducted and found that LINC02474 restrained *GZMB* expression to suppress the proliferation and metastasis of colorectal cancer cells (Tiantian et al., 2021). This had also been reported in BC and the following observations indicated that the expression of *GZMB* in invasive BC was 4.910 times higher than that in normal tissues (Finak et al., 2008). Interestingly, our study suggested that *GZMB* acted as a tumor suppressor in BC, and its expression was significantly decreased in the high-risk group, which was consistent with previous results.

Inflammasome activation also initiates a programmed cell death termed pyroptosis, then ends to the release of cell contents and many inflammatory factors, such as IL-1β and Interleukin-18 (IL-18). *IL-18* is a pleiotropic pro-inflammatory cytokine belonging to the IL-1 superfamily, playing an essential role in inflammatory responses and cancers (Maryam and Abbas, 2017). It was found that IL-18 released by inflammasomes protected GC cells from apoptosis to stimulate the progression (Virginie et al., 2018). *IL-18* levels inversely correlated with patient prognosis and lower IL-18 levels were observed in surviving patients (Nakamura et al., 2018). Further study on the mechanism of *IL18* in PDAC showed that Pin1 promoted the proliferation and progression of pancreatic cancer cells by increasing the expression of *IL-18* and continuously activating NF-kB cell pathway (Sun et al., 2020). However, in this study, we found that the expression of *IL18* was significantly decreased in the high-risk group, suggesting that *IL18* might act as a tumor suppressor to prevent the progression of BC, which was controversial with previous research. Therefore, it was possible that *IL18* held multiple roles in different tumors, and our findings provided an insight for the investigation of *IL18*-mediated carcinogenesis and development of BC.

Interferon regulatory factor (*IRF*)-1, also called interferon regulatory factor 1, is encoded by the *IRF1* gene in humans (Komatsu et al., 2016). *IRF1* is the first member of the IRF family of transcription factor and widely expressed in various tissues, capable of activating or repressing the transcription of multiple target genes (Martinović et al., 2015). In addition to the function of transcription factor, *IRF1* could also activate the expression of tumor suppressor p53 and pyroptosis key factor *GSDMD*, and regulate various types of cell death under different experimental conditions, including apoptosis, pyroptosis, and necroptosis (Ratana et al., 2016). It was found that the expression of *IRF1* in CRC was significantly lower than that in normal tissues. Overexpression of *IRF1* could inhibit the proliferation, migration, and metastasis of CRC cells. Further study on the mechanism showed that *IRF1* mediated the anticancer effect by inhibiting the RAS-RAC1 pathway (Min et al., 2019). IRF1 also played a role of tumor suppressor gene in non-small cell lung cancer (NSCLC). The expression of *IRF1* in NSCLC was significantly lower than that in normal tissues, and the expression level was positively correlated with prognosis which inhibited the proliferation of NSCLC by negatively regulating the expression of carcinogenic KPNA2 under growth stimulation and hypoxia (Min et al., 2019). *IRF1* also had a tumor inhibitory effect in BC. The results showed that the low expression of IRF1 in BC patients was closely related to the risk of recurrence and death. Overexpression of *IRF1* could significantly reduce the occurrence of human BC xenografts (Cavalli et al., 2010). Although *IRF1* was currently involved in the occurrence and development of many kinds of tumors, it was rare to promote or inhibit tumors through the influence of pyroptosis. A study on CRC had found that IRF1 could mediate a variety of cell death modes such as apoptosis, necrosis, and pyroptosis, which included that *IRF1* could promote the expression of GSDMD to induce cell pyroptosis and inhibit CRC proliferation (Rajendra et al., 2020). Our study found that *IRF1* was in favor of good prognosis in BC, but the relationship between *IRF1*-mediated cell death and the occurrence and development of BC and other tumors had not been elucidated in detail. Further research was needed to clarify the mechanism in the future.

Similar with *GZMB*, *GZMA* is a member of the serine protease family. *GZMA* cleavaged and activated *GSDMB* to induce target cells pyroptosis produced by cytotoxic lymphocytes. This immune effect mechanism promoted CTL-mediated tumor clearance, which was traditionally considered to be an antineoplastic drug (Zhiwei et al., 2020). However, contrary results had been observed in CRC. The expression of *GZMA* in CRC was positively correlated with inflammatory reaction and malignance, which suggested that *GZMA* was involved in the malignant progression of colorectal cancer. Further exploring the mechanism showed that *GZMA* enhanced the inflammatory response and mediated tumor progression by inducing the production of IL6 in macrophages and activating pSTAT3 pathway in cancer cells (Llipsy et al., 2020). In our study, *GZMA* functioned as a tumor suppressor, which was positively correlated with the prognosis of BC, but whether it affected the occurrence and development of BC through cell pyrolysis still needs to be confirmed *in vivo* and *in vitro*.

Pyroptosis was found to play a dual role in tumor occurrence and development, by participating in tumorigenesis and anti-tumor immunity at all stages of tumor development. On the one hand, long-term chronic inflammation can promote the development of a tumor; on the other hand, the sudden activation of pyroptosis will lead to the infiltration of a variety of immune cells and inhibit tumor progression (Galluzzi et al., 2017). Functional enrichment analysis of DEGs in high- and low-risk groups, we also found that these DEGs were mainly involved in inflammatory response and immune response, suggesting that pyroptosis could regulate inflammatory response and immune response. Based on the close relationship between pyroptosis and tumor immunity, we found that the proportion of anti-tumor immune cells in the low-risk group was significantly higher than that in the high-risk group, suggesting that the immune function of the high-risk group was impaired as a whole, while surprisingly, the proportion of Treg cells in the low-risk group was also significantly higher, which should be further studied on the regulation of TREG cells in the tumor microenvironment of BC. At the same time, the immune activation pathway in the low-risk group scored more than that in the high-risk group, which was also verified in the GEO dataset. Combined with the above results, the poor prognosis of high-risk BC might be caused by low anti-tumor immunity. Except for that, we also ranked the immune score in the high- and low-risk group and found that the immune score and matrix score in the low-risk group were significantly higher than those in the high-risk group, while the tumor purity in the low-risk group was lower. In line with previous studies, patients with high-risk BC had higher tumor purity, lower immune levels, and poor prognosis. It is worth noting that tumor purity refers to the proportion of tumor cells in tumor tissue, which is composed of immune cells, stromal cells, and so on. Studies have found that tumor purity is significantly related to the clinical characteristics, genome expression, and biological characteristics of tumor patients. Ignoring the influence of tumor purity can lead to systematic bias in the process of tumor genotyping, risk of recurrence, and prediction of curative effect (Dvir et al., 2015). Nevertheless, tumor purity largely depends on the sampling procedures, and the purity of standard tumor surgical samples is usually less than 70%. As a result, tumor purity cannot be used as a good indicator of tumor classification and prognostic markers. In terms of our established risk model, it was related to tumor purity to a certain extent, but there were still great obstacles to applying it to the clinic and more in-depth research and detection methods are needed in the future. Therefore, our research results are only based on the TCGA database, which can only explain the correlation between each other to a certain extent and provide clues for further research.

In recent years, major advances have been made in cancer immunotherapy, thereby drastically improving the prognosis of cancer patients. The combination that integrates immunotherapy with chemotherapy, radiation therapy, and targeted molecular therapy benefits from different molecular types of BC. Nevertheless, biomarkers or models that provide accurate prognosis predictions are still lacking. Currently available biomarkers to predict immunotherapy efficacy mainly include TMB and microsatellite instability. TMB is referred to as a whole number of gene variants. Clinical data demonstrated that patients with high TMB were more likely to benefit from immune checkpoint inhibitor therapy, which suggested that TMB should be an appropriate biomarker for assessing the effect of immune treatment (Rizvi et al., 2015). MSI is a condition of genetic hypermutability that results from impaired DNA MMR function, which has been proven to be one of the valuable biomarkers for the clinical efficacy of anti-PD-1/PD-L1 immune checkpoint inhibitors (Dudley et al., 2016). Therefore, in this study, we analyzed the relationship between the four core genes in the risk model, risk score, and MSI and TMB. It was found that the expression levels of *GZMA*, *GZMB*, *IL18,* and *IRF1* were positively correlated with TMB, suggesting that these four PRGs might be potential predictors of TMB. At the same time, we also found that the level of TMB in the low-risk group was significantly higher than that in the high-risk group, indicating that the benefit of immunotherapy in the low-risk group was better than the high-risk group. Then we observed that the high expression of *GZMA*, *GZMB*, *IL18*, and *IRF1* promoted the efficacy of immune checkpoint blocking therapy and effectively improved the prognosis of patients. Eventually, we verified the expression of model genes in clinical samples and explored the link between inflammation and pyroptosis in BC to a certain extent. In BC, LPS was used to activate the NF-kB signaling pathway, causing cellular inflammation, inducing the occurrence of pyroptosis. Meanwhile, we detected the expression level of IL18 and found that the expression level was significantly increased, and it had also been verified to be overexpressed in BC tissue based on the TCGA cohort, indicating that there was a close relationship between cellular inflammation, pyroptosis, and BC, and further studies are needed to clarify the mechanism.

In summary, we constructed a PRG-based prognostic model to systematically evaluate the prognosis of BC patients, which had robust predictive ability. We also proved that there was a strong relationship between pyroptosis and the occurrence and development of BC, and the expression of PRGs influenced the progression of cancer. In addition, we also found that PRGs were involved in regulating the composition of the immune microenvironment and the efficacy of immunotherapy in BC, which provided an important basis for further study of PRGs and immune function and immunotherapy targets of patients.

## Data Availability

The data could be downloaded at TCGA, GEO and ICGC, and the code used during the current study are available from the corresponding author on reasonable request.

## Abbreviations

BC, breast cancer; CTL, cytotoxic T lymphocytes; DEGs, differentially expressed genes; GC, gastric cancer; GEO, gene expression omnibus; GO, gene ontology; GSDMD, gasdermin D; GSDME, gasdermin E; GZMB, granzyme B; HC, hepatocellular carcinoma; ICB, immune checkpoint blockers; IL-18, Interleukin-18; IRF, interferon regulatory factor; KEGG, Kyoto Encyclopedia of Genes and Genomes; LASSO, least absolute shrinkage and selection operator; MSI, microsatellite instability; NK, natural killer; NSCLC, non-small cell lung cancer; OS, overall survival; PPI, protein-protein interaction; PRGs, pyroptosis-related genes; ssGSEA, single-sample gene set enrichment analysis; STRING, Search Tool for the Retrieval of Interacting Genes; TCGA, The Cancer Genome Atlas; Th, T helper; TIDE, tumor immune dysfunction and exclusion; TILs, tumor-infiltrating lymphocytes; TMB, tumor mutation burden; Treg, regulatory T.
