# Supplementary figures and images for: A Pyroptosis-Related Gene Signature Predicts Prognosis and Immune Microenvironment for Breast Cancer Based on Computational Biology Techniques
Source: Front Genet. 2022 Apr 7;13:801056. doi: 10.3389/fgene.2022.801056 (PMC9021921; doi:10.3389/fgene.2022.801056)

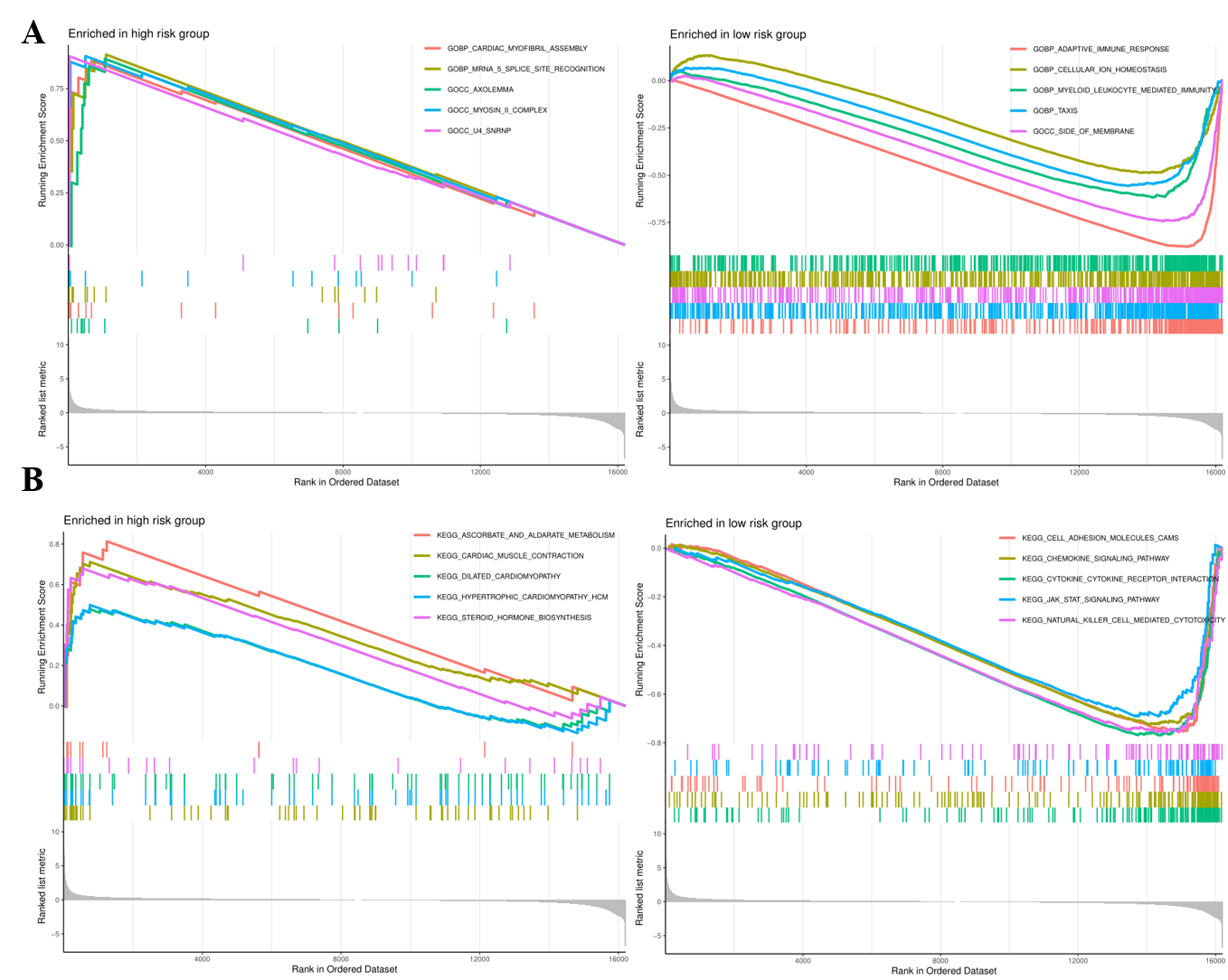

Supplement: Supplementary file 1 [file Image6.TIF]

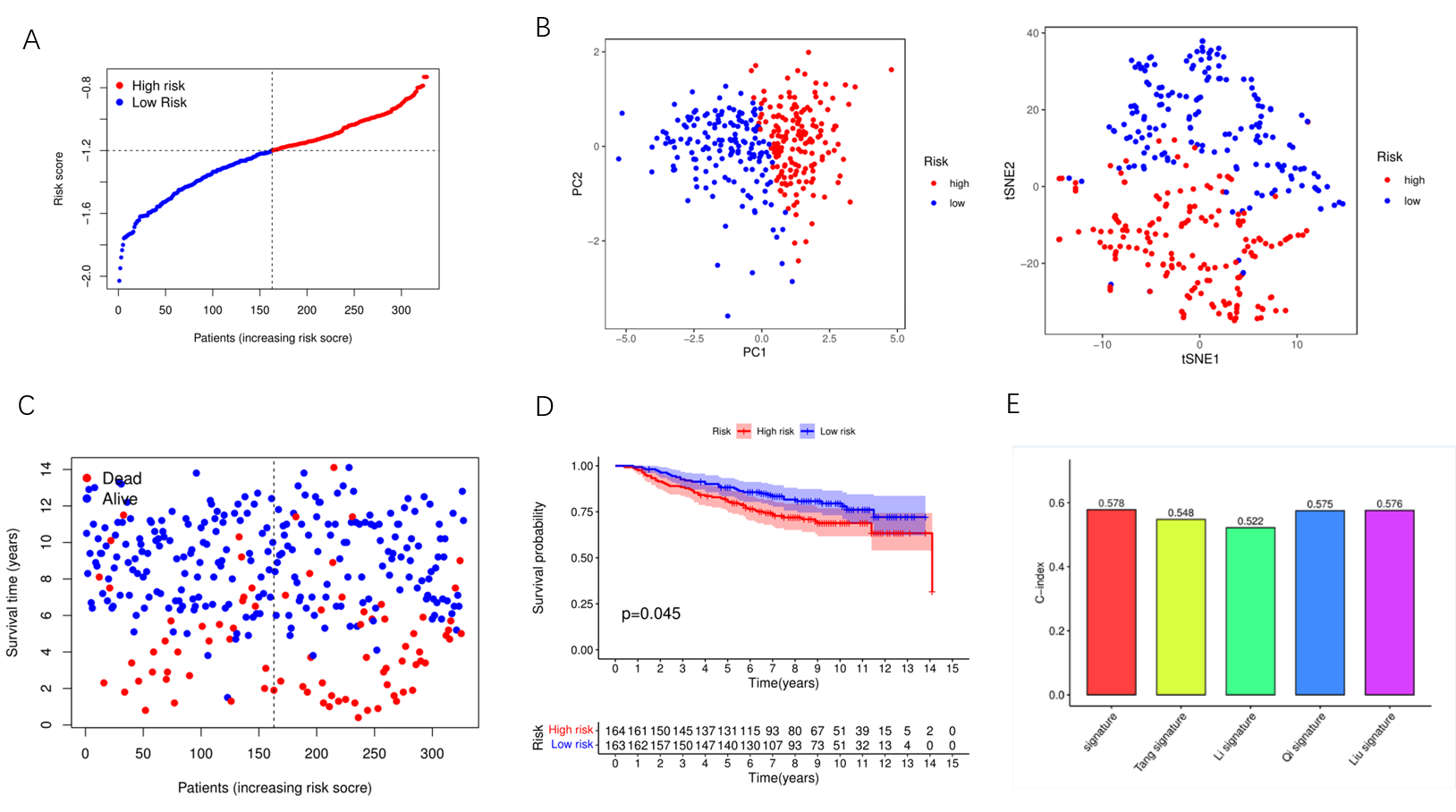

Supplement: Supplementary file 3 [file Image3.TIF]

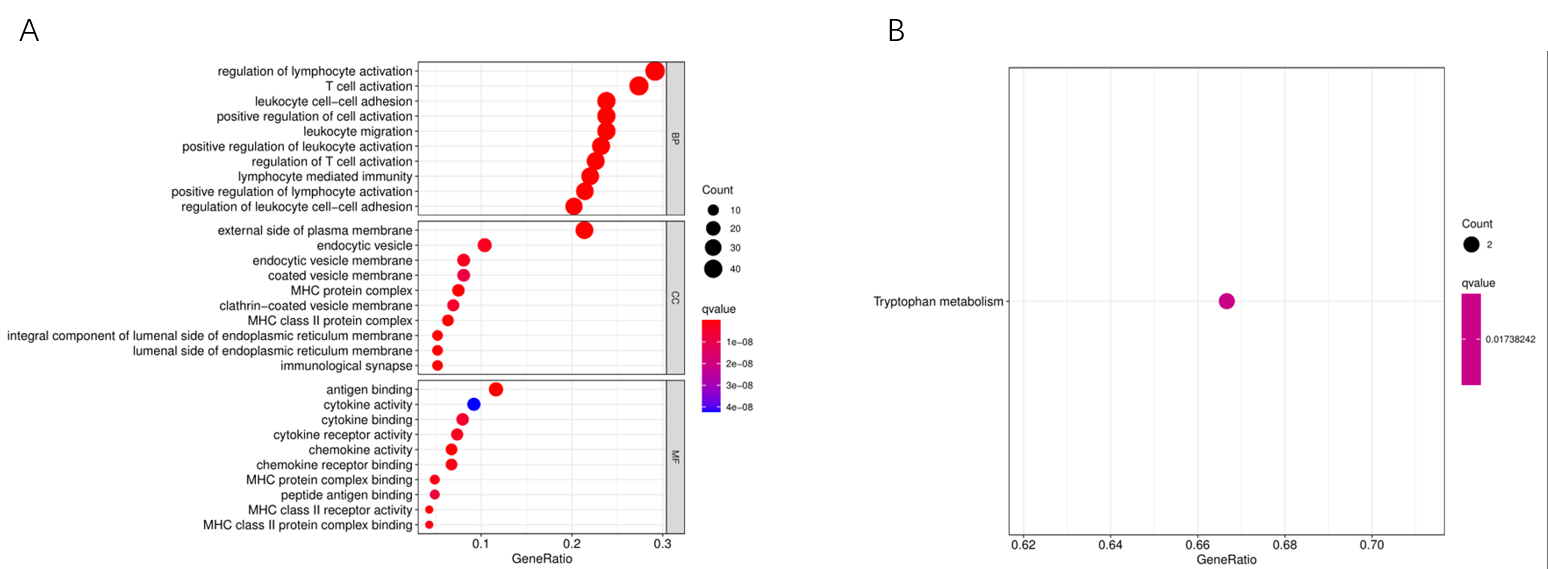

Supplement: Supplementary file 4 [file Image4.TIF]

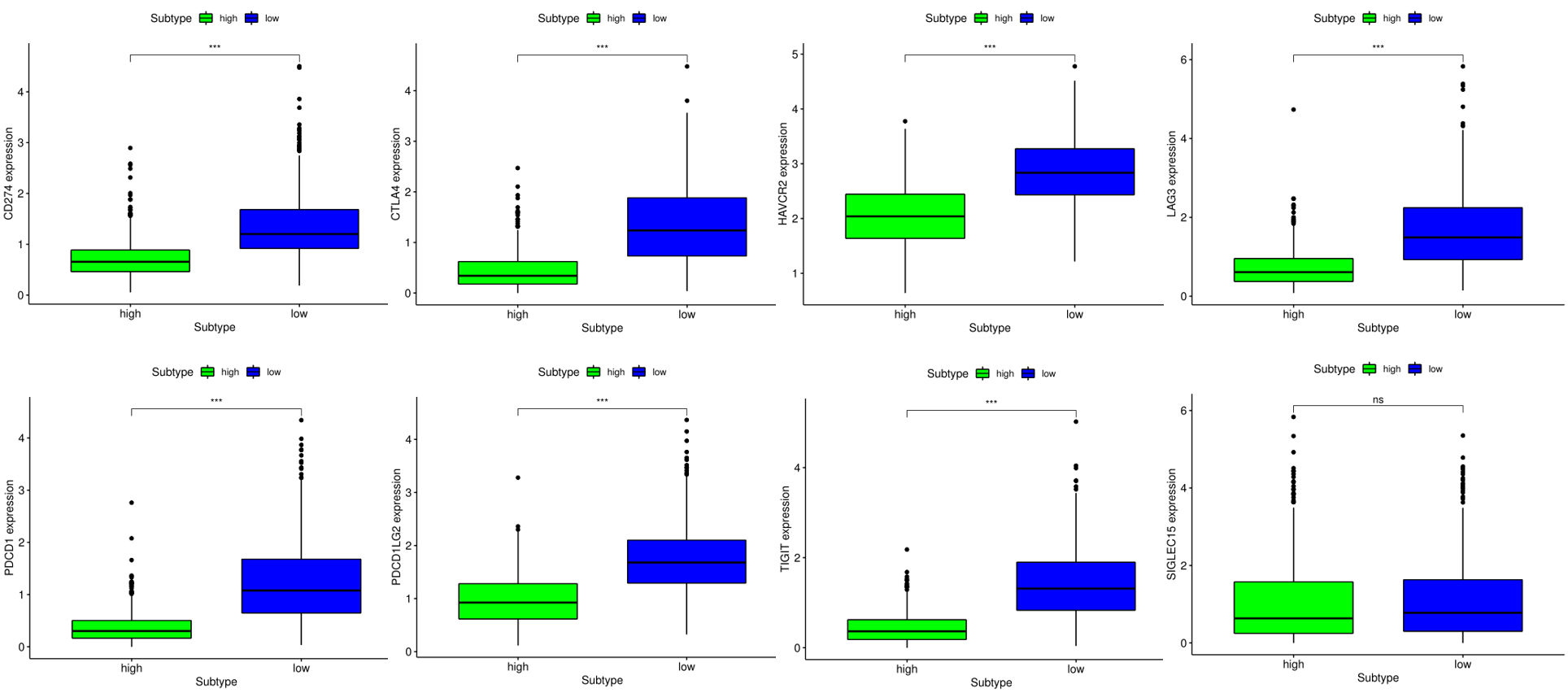

Supplement: Supplementary file 5 [file Image9.TIF]

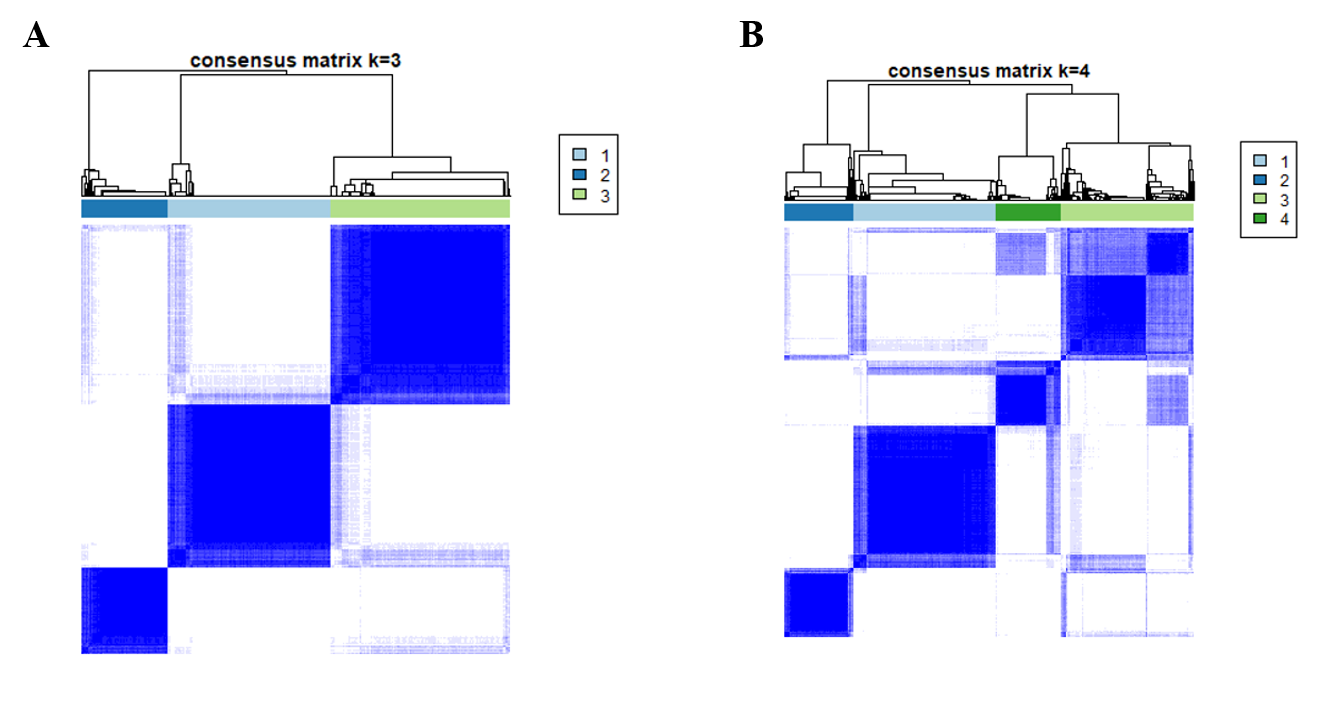

Supplement: Supplementary file 6 [file Image2.TIF]

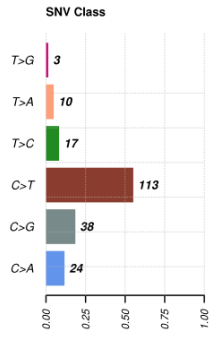

Supplement: Supplementary file 7 [file Image1.TIF]

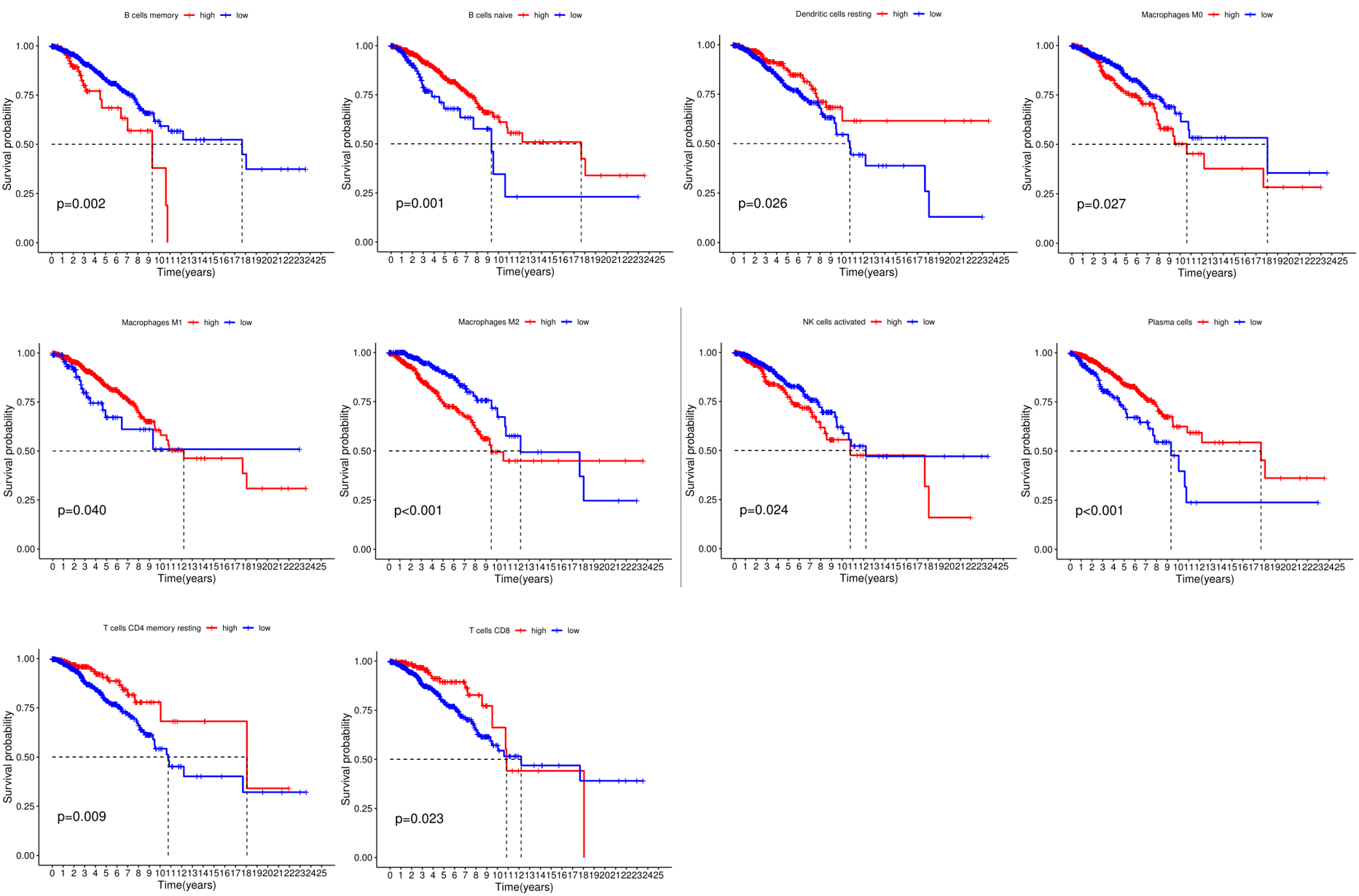

Supplement: Supplementary file 8 [file Image7.TIF]

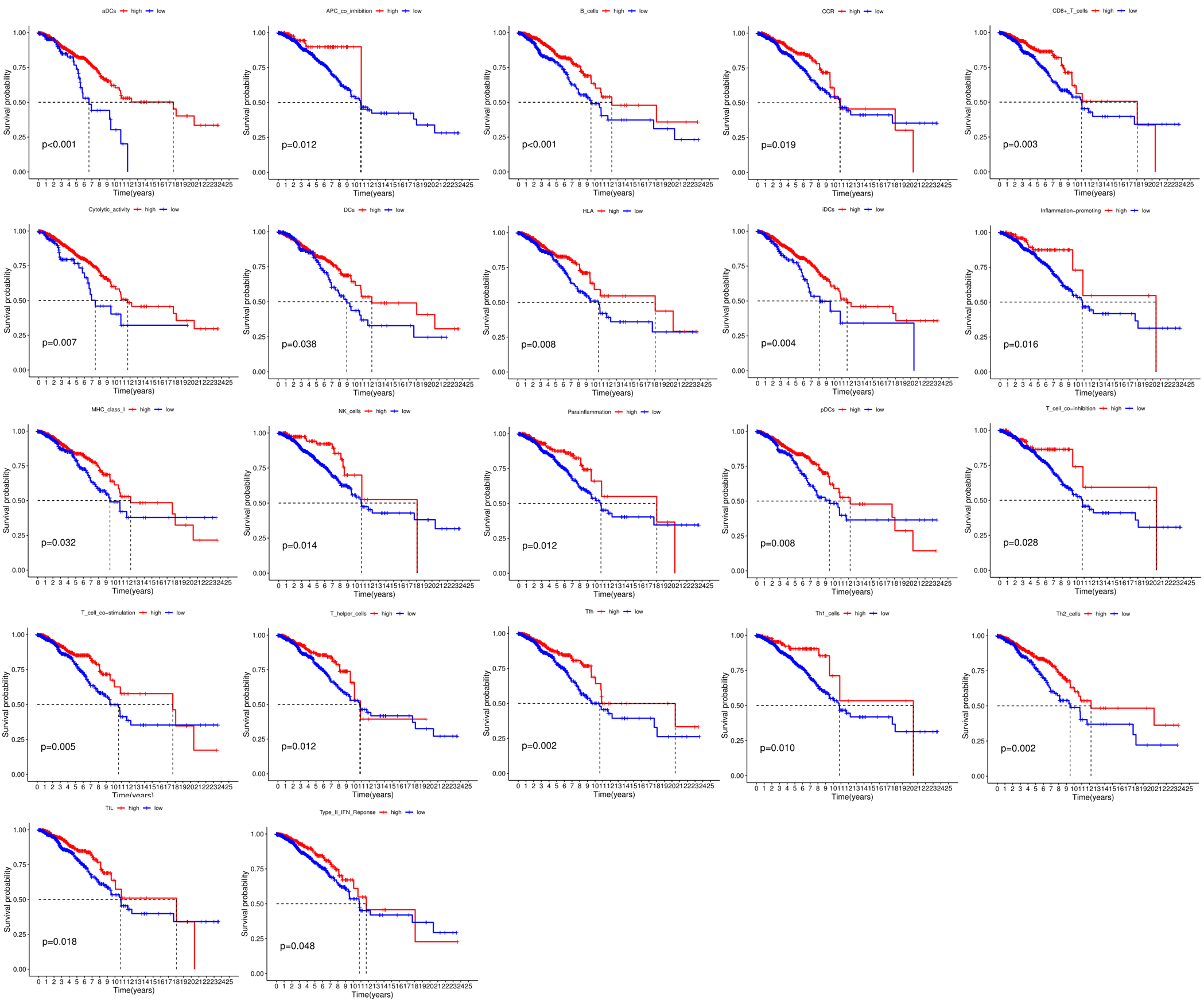

Supplement: Supplementary file 9 [file Image8.TIF]

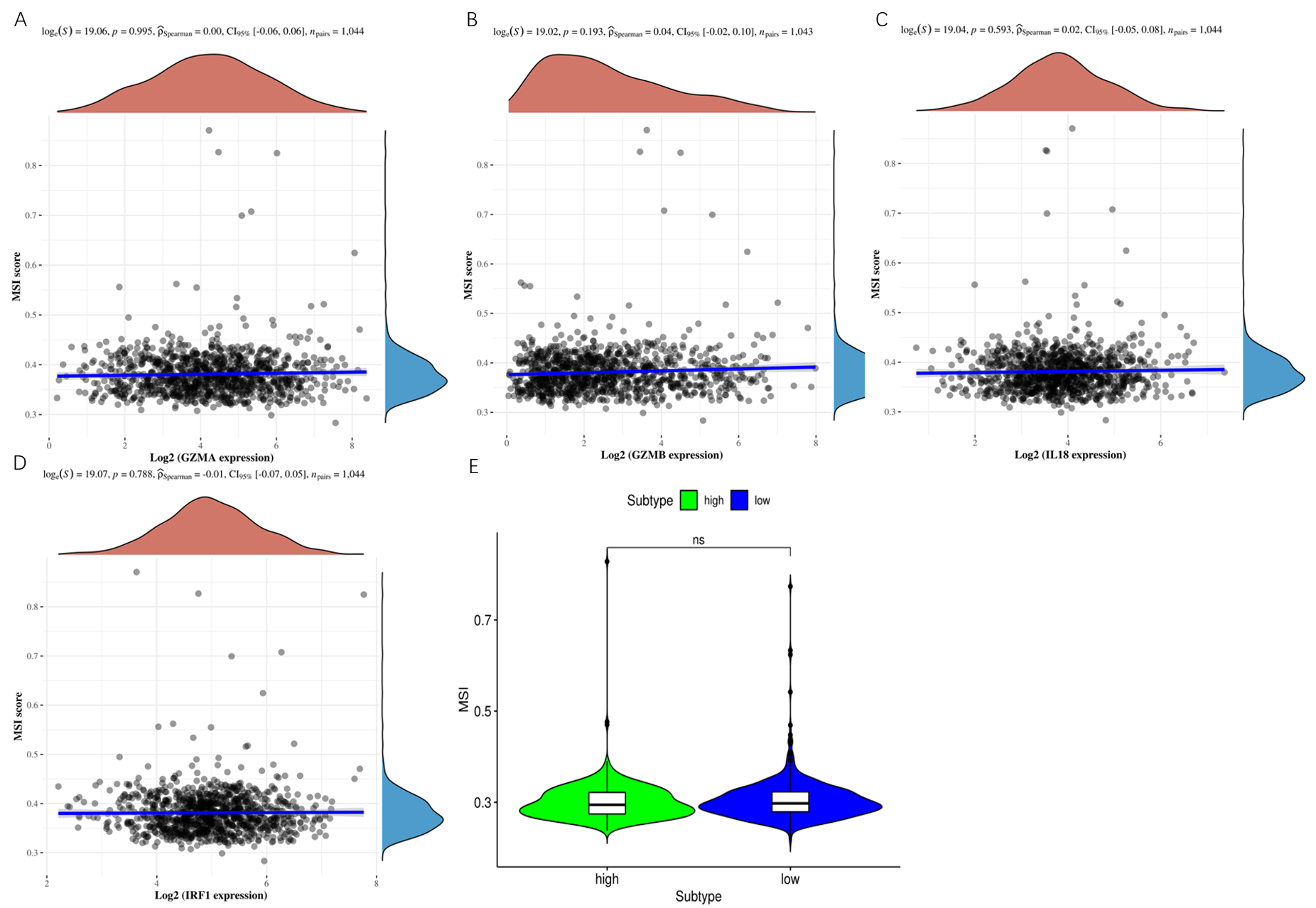

Supplement: Supplementary file 10 [file Image5.TIF]
