# Supplementary material for: A Pyroptosis-Related Gene Signature Predicts Prognosis and Immune Microenvironment for Breast Cancer Based on Computational Biology Techniques
Source: Front Genet. 2022 Apr 7;13:801056. doi: 10.3389/fgene.2022.801056 (PMC9021921; doi:10.3389/fgene.2022.801056)
Supplement: Supplementary file 2 [file Table1.docx]

Supplementary table 1 Clinicopathological parameters of patients

| Case id | Age | Gender | Tumor size (cm) | T | N | M | Grade | Type |
| --- | --- | --- | --- | --- | --- | --- | --- | --- |
| 1 | 54 | Female | 2.6*2.4*2 | 2 | 0 | 0 | III | Invasive carcinoma |
| 2 | 37 | Female | 3*3 | 2 | 1 | 0 | NA | Invasive ductal carcinoma |
| 3 | 35 | Female | 3*2*1.5 | NA | NA | NA | III | Invasive carcinoma |
| 4 | 39 | Female | 6*4*3.5 | 3 | 2 | 0 | III | Invasive carcinoma |
| 5 | 42 | Female | 2.4*1.6 | 2 | 2 | 0 | II | Invasive ductal carcinoma |
| 6 | 64 | Female | NA | 4b | NA | 1 | III | Invasive ductal carcinoma |
| 7 | 51 | Female | 7*4 | 3 | 1 | NA | III | Invasive ductal carcinoma |
| 8 | 57 | Female | NA | 4 | 3 | 1 | NA | Invasive carcinoma |
| 9 | 47 | Female | NA | NA | 1 | 0 | IV | Invasive carcinoma |

NA: Unknown
