# Supplementary material for: A Pyroptosis-Related Gene Signature Predicts Prognosis and Immune Microenvironment for Breast Cancer Based on Computational Biology Techniques
Source: Front Genet. 2022 Apr 7;13:801056. doi: 10.3389/fgene.2022.801056 (PMC9021921; doi:10.3389/fgene.2022.801056)
Supplement: Supplementary file 11 [file DataSheet1.DOCX]

**Figure 1 Identification of 38 differentially expressed pyroptosis-related genes and the interactions among them.**

A: Heatmap of the differential pyroptosis-related gene expression between normal and tumor samples (*: *P*<0.05, **: *P*<0.01, ***: *P*<0.001).

B: PPI network showing the interactions of the PRGs (interaction score = 0.4)

C: The correlation network of the pyroptosis-related genes

**Figure 2 Somatic alterations of PRGs in BC**

A: Summary of the mutation in TCGA

B: Waterfall of the mutation in TCGA (The upper pane refers to the frequency of somatic PRGs mutations in each patient in the 149 patients; The lower pane refers to the somatic mutation frequency of one PRG in all 149 patients)

**Figure 3 Tumor classification based on the pyroptosis-related DEGs.**

A: 1076 BC patients were grouped into two clusters according to the consensus clustering matrix (k = 2)

B: Heatmap and the clinicopathological characters of the three clusters classified by these DEGs

C: Kaplan–Meier OS curves for the two clusters

**Figure 4 Establishment of a risk model based on TCGA cohort**

A: A forest plot consists of four OS-related genes via univariate cox analysis

B, C, D, E and F: A four OS-related genes signature was constructed through LASSO, SVM and random forest

G: The distribution and median value of the risk scores

H: PCA and t-NSE plots for patients based on the risk score

I: The distributions of OS status in each patient

J: Kaplan–Meier curves for the OS of patients in the high- and low-risk groups

**Figure 5 Independent prognostic value of the risk model**

A: Univariate analysis in the TCGA cohort

B: Multivariate analysis in the TCGA cohort

C: Univariate analysis in the GEO cohort

D: Multivariate analysis in the GEO cohort

E: Heatmap for the connections between clinicopathological features and the risk groups

F: Construction of a nomogram to predict survival of patients based on clinical parameters and risk score in TCGA cohort

G: Construction of a nomogram to predict survival of patients based on clinical parameters and risk score in GEO cohort

H: Calibration curves for the nomogram based on TCGA cohort

I: Calibration curves for the nomogram based on GEO cohort

**Figure 6: Immune microenvironment between high and low risk group patients in TCGA and GEO cohort**

A: Comparison of the enrichment scores of 16 types of immune cells between high risk and low risk groups in TCGA

B: Comparison of the enrichment scores of 13 immune-related pathways between high risk and low risk groups in TCGA

C: Comparison of the enrichment scores of 16 types of immune cells between high risk and low risk groups in GEO

D: Comparison of the enrichment scores of 13 immune-related pathways between high risk and low risk groups in GEO

E: The relationship between tumor purity and risk score in TCGA cohort

F: The relationship between stromal score and risk score in TCGA cohort

G: The relationship between immune score and risk score in TCGA cohort

H: The relationship between estimate score and risk score in TCGA cohort

**Figure 7: TMB, MSI, and ICB analysis of PRG in TCGA and ICGC cohort**

A: The correlation between GZMA and TMB in TCGA

B: The correlation between GZMB and TMB in TCGA

C: The correlation between IL18 and TMB in TCGA

D: The correlation between IRF1 and TMB in TCGA

E: The correlation between risk score and TMB in TCGA

F: The distribution of immune response scores between high and low expression of GZMA in ICGC

G: The distribution of immune response scores between high and low expression of GZMB in ICGC

H: The distribution of immune response scores between high and low expression of IL18 in ICGC

I: The distribution of immune response scores between high and low expression of IRF1 in ICGC

**Figure 8: The enriched immune cells and immune functions between high- and low- risk groups via CIBERSORT**

**Figure 9: Evaluation and comparison of the TIDE score between high- and low- risk groups**

A: The comparisons of the Dysfunction, Exclusion, MSI and TIDE scores between high- and low- risk groups

B: The comparison of the predictive efficiency between the model and TIDE, TIS scores

**Figure 10: The expression validation of the candidate genes**

A: The Kaplan-Meier curves of the BC patients between high and low expression of the candidate genes

B: The expression of the four candidate genes between tumor and normal tissues

C: The mRNA expression of IL 18 after incubating via different concentrations of LPS in MCF-7

**Supplementary figure 1**

The SNV class of the mutation spectrum in BC patients

**Supplementary figure 2**

A: BC patients were grouped into three clusters according to the consensus clustering matrix (k = 3)

B: BC patients were grouped into four clusters according to the consensus clustering matrix (k = 4)

**Supplementary figure 3: Validation of a risk model based on GEO cohort**

A: Distribution of patients in the GEO cohort

B: PCA and t-NSE plot for the distribution of the patients

C: The survival status for each patient

D: Kaplan–Meier curves for comparison of the OS between low- and high-risk groups

E: The C-index of the risk model and other established signatures

**Supplementary figure 4: Functional analysis based on the DEGs in the TCGA cohort.**

A: Bubble plot for GO enrichment analysis

B: Bubble plot for KEGG enrichment pathway analysis

**Supplementary figure 5**

A: The correlation between GZMA and MSI in TCGA

B: The correlation between GZMB and MSI in TCGA

C: The correlation between IL18 and MSI in TCGA

D: The correlation between IRF1 and MSI in TCGA

E: The correlation between IRF1 and MSI in TCGA

**Supplementary figure 6: GSEA analyses between high- and low- risk groups**

A: The GO analysis results enriched in high- and low- risk groups

B: The KEGG analysis results enriched in high- and low- risk groups

**Supplementary figure 7**

The Kaplan-Meier curves of the BC patients between high- and low- level immune cells

**Supplementary figure 8**

The Kaplan-Meier curves of the BC patients between high- and low- level immune functions

**Supplementary figure 9**

The expression level of the immune checkpoint molecules between high- and low- risk groups
